# A risk assessment indicator system for common diseases in children and adolescents

**DOI:** 10.1371/journal.pone.0351870

**Published:** 2026-06-17

**Authors:** Yun Shao, Bohan Hu, Yuting Lu, Shuo Yu, Ji Liang

**Affiliations:** 1 School of Public Health, Fudan University, Shanghai, China; 2 Shanghai Putuo District Center for Disease Control and Prevention, Shanghai, China; 3 NHC Key Laboratory of Health Technology Assessment (Fudan University), Shanghai, China; SRM Institute of Science and Technology, INDIA

## Abstract

**Background:**

Adolescence is a critical stage for healthy development. Currently, common diseases such as myopia, obesity, dental caries, and spinal curvature abnormalities are highly prevalent among children and adolescents, with an obvious trend of younger onset. These diseases are caused by the complex interaction of multi-dimensional factors. However, existing interventions are mostly limited to individual diseases, lacking systematic prevention and control tools. Therefore, there is an urgent need to construct a scientific evaluation index system for health-influencing factors.

**Objective:**

Targeting the four key common diseases emphasized in China’s national health policies – myopia, obesity, dental caries, and spinal curvature abnormalities – among children and adolescents aged 7–19 years, this study constructed a multi-dimensional risk assessment indicator system to support evidence-based prevention and policy-making for this population.

**Methods:**

Guided by the National Program of Action for Children’s Development in China (2021–2030) and the “Healthy China 2030” Planning Outline, a three-level indicator framework was initially formulated through literature research and expert meetings. The Delphi expert consultation method was adopted for two rounds of screening and optimization of indicators. Experts scored each indicator on a scale of 1–5 in terms of importance, feasibility, and sensitivity, with indicators excluded if their mean score was < 3.5 and coefficient of variation >0.3.

**Results:**

The effective return rate of the two-round Delphi is 100.0%, the mean expert familiarity coefficient (Cs) was 0.83, and the mean judgment coefficient (Ca) was 0.93, and the expert authoritative coefficient (Cr) value is 0.88. In the second round of consultation, the importance coordination coefficient is 0.284, the feasibility coordination coefficient is 0.185, and the sensitivity coordination coefficient is 0.314, all of which are statistically significant (*P* < 0.001). This indicator system consists of a total of 55 three-level indicators.

**Conclusion:**

Validated through two rounds of expert deliberation, the indicator system demonstrates sound scientific rigor. The preliminary development of a risk assessment indicator system for common diseases in children and adolescents can be used for the measurement of the level of factors affecting the health of common diseases in children and adolescents after its reliability and validity are verified in practical applications.

## Introduction

Children and adolescents represent a critical period for healthy development across the entire life course [1].The World Health Organization (WHO) has included childhood myopia, dental caries, and obesity in its non-communicable disease prevention and control framework [2], and has released documents such as the World Report on Vision [3] and the Global strategy and action plan on oral health 2023–2030 [4] to address the high prevalence and increasingly younger age of onset of these conditions. Accordingly, National Health Commission of the People’s Republic of China has formulated the “Five-Health” Promotion Action Plan for Children and Adolescents (2026–2030) [5], which identifies obesity, myopia, dental caries, and spinal curvature abnormalities as key priority areas for prevention and control.

From the perspective of specific data, according to WHO data in 2022, 390 million children and adolescents aged 5–19 years had overweight, including 160 million with obesity [6]. A population-based study demonstrated that the overall prevalence of myopia among children and adolescents in Wuhu, China, was 56.92% [7], with a particularly rapid increase in prevalence among those aged 10–18 years [8]. Similarly, a cross-sectional survey of 16,199 primary and secondary school students in Henan Province in 2019 showed that the overall prevalence of dental caries has risen from 39.75% in 2010 to 53.21%, indicating that the problem of caries in children is not only widespread but also on an increasing trend [9]. A provincial surveillance study in Jiangsu, China reported an overall detection rate of 2.1% for spinal curvature abnormalities among children and adolescents during 2021–2023, highlighting this condition as an important public health issue [10].

A substantial body of evidence confirms that conditions like obesity [11,12] and myopia [13] result from the complex interplay of social, environmental, behavioral, and health services factors. These diseases confer both immediate and long-term health consequences. For example, a large cross-sectional study based on nationally representative data of U.S. children and adolescents showed that, compared with those with mild-to-moderate obesity (body mass index [BMI] ≥ 95th percentile to <160% of the 95th percentile), individuals with extremely severe obesity (BMI ≥ 160% of the 95th percentile) had a 4.94-fold higher risk of prediabetes/diabetes and a 6.74-fold higher risk of metabolic dysfunction–associated steatotic liver disease [14]. A large life-course cohort study [15] has provided compelling evidence for the long-term health impacts of childhood obesity. Compared with children whose childhood BMI was between the 15th and 50th percentiles, those in the obese group (≥99th percentile) had hazard ratios (HRs) of 2.00 and 1.68 for developing endocrine/metabolic diseases and circulatory system diseases in adulthood. Childhood and adolescent obesity [16] not only harms the current physical health, causing chronic diseases such as diabetes, hypertension, but also increases the risk of cardiovascular diseases in adults. However, existing intervention strategies are mostly limited to individual diseases, and there is a lack of systematic and comprehensive prevention and control models targeting common health determinants.

In this regard, this research has developed a risk assessment indicator system for common diseases (obesity, myopia, dental caries, and spinal curvature abnormalities) in children and adolescents, covering five dimensions: social determinants, school environment, health services, genetic and early life factors, and health-related behavioral factors, which can provide reference for identifying key health determinants affecting these common diseases in children and adolescents, thereby providing an evidence-based foundation for developing integrated interventions to ultimately improve the overall health of children and adolescents.

## Materials and methods

**Subjects.** Delphi experts were selected based on the following criteria: (1) Professional Field: Experts should have rich practical experience in fields such as Child & Adolescent Health, Maternal and Child Health (MCH), Pediatrics, Preventive Medicine, Health Management, and Nursing. (2) Academic Level: They should possess a relatively high academic level in relevant fields and have a certain professional influence in the industry. (3) Title and Qualifications: They need to hold an associate senior or above professional title, or have an equivalent management rank. (4) Work Experience: They should have at least 11 years of work experience in related fields, with solid industry accumulation and practical knowledge. Potential experts were initially identified through recommendations from collaborative institutions and peers. After eligibility verification, consultation questionnaires were sent via email. Ultimately, a total of 16 experts from Centers for Disease Control and Prevention, universities, hospitals, maternal and child health centers, health commissions, primary/secondary schools, and community health service centers were enrolled in this study.

### Method preliminary index system

The first-level and second-level indicators were classified based on national policies and also with reference to the life course perspective-based adolescent health assessment framework proposed by Tao FB (2014) [17]. Guided by core policy documents including the National Program of Action for Children’s Development in China (2021–2030) and the Healthy China 2030 Planning Outline, this study integrated the life course perspective and adolescent health conceptual framework to divide the content of children and adolescents’ health monitoring into 4 first-level indicators and 8 second-level indicators. The construction of third-level indicators was mainly based on literature review, existing monitoring systems, and the needs of expert consensus. A structured literature review was conducted across Chinese and English databases (e.g., CNKI, Wanfang Data, PubMed) and official government websites, using keywords such as “children,” “adolescents,” “students,” “myopia,” “overweight,” “obesity,” “dental caries,” “spinal curvature abnormalities,” “Shanghai,” “intervention,” “management,” “evaluation,” “Myopia,” “Students,” “Adolescents,” “Children,” “Oral,” “Scoliosis,” “Caries,” “Shanghai,” and “Obesity.” By incorporating mature indicators from the Shanghai Student Common Diseases and Health Influencing Factors Monitoring and Intervention Scale, a total of 54 third-level indicators were finally formulated.

### Form a pool of index items

Building on these preliminary efforts, the research team integrated policy requirements, theoretical frameworks, literature evidence, and existing monitoring indicators to establish an evaluation indicator inventory comprising 4 first-level indicators, 8 second-level indicators, and 54 third-level indicators. This inventory covers health determinants, health status, and relevant health work related to overweight and obesity, myopia, dental caries, and spinal curvature abnormalities in children and adolescents. Based on this inventory, an expert consultation questionnaire was designed for subsequent Delphi method implementation or expert consultation procedures.

### Indicator score assignment

At the time of correspondence, experts evaluate the importance, sensitivity and feasibility of the indicator, and score it according to 1–5 points. According to the experts’ familiarity with the indicators and their judgment basis, through the experts’ self-evaluation, the expert familiarity coefficient (Cs) of the indicators is calculated. The expert’s familiarity with the indicators is as follows: Cs = 1.0 is very familiar, Cs = 0.8 is familiar, Cs = 0.6 is average, Cs = 0.4 is not familiar, Cs = 0.2 is unfamiliar. The basis of judgment and the degree of influence assigned are shown in Table 1.

**Table 1:** Quantification of the basis for judgment and level of impact.

Basis for Judgment	Level of Impact				
	Very High	High	Medium	Low	Very Low
Theoretical Analysis	0.30	0.24	0.18	0.12	0.06
Practical Experience	0.40	0.32	0.24	0.16	0.08
Understanding of Domestic and International Peers	0.20	0.16	0.12	0.08	0.04
Intuition	0.10	0.08	0.06	0.04	0.02

### Indicator modification

Indicators were deleted or revised if the mean score for importance, feasibility, or sensitivity was less than 3.5 or the coefficient of variation exceeded 0.30, combined with qualitative expert feedback and group discussions. In each round of consultation, experts could propose modifications, additions, or deletions to the indicators, and a new consultation form will be formed after incorporating the opinions of experts. Feedback the anonymous opinions put forward in the previous round to each expert, and carry out the next round of evaluation and screening.

**Statistical analysis.** Using Excel 2016 and SPSS 24.0 software for data entry and statistical processing, *P* < 0.05 is a statistically significant difference.

**Ethical statement and informed consent.** The core component of this study was an expert consultation. The first page of the consultation questionnaire included a formal invitation letter that clearly specified the research objectives, scope of the consultation, and other relevant information. Experts were informed of the voluntary nature of their participation and their right to withdraw from the study at any time. Oral informed consent was obtained from all experts prior to their engagement in the consultation. We sent expert consultation questionnaires via email. When experts replied and completed the questionnaires, this was regarded as their informed consent to voluntarily participate in the study. Questionnaires were distributed via email, and completed responses were returned by the same means. This study solely collected expert opinions and did not involve human or animal experiments, medical interventions. All information obtained was used exclusively for the purposes of this study and was treated with strict confidentiality, with no disclosure to third parties. Upon completion of the consultation, participating experts received a reasonable honorarium, which was disbursed through bank transfer. All transaction records were retained to ensure the compliance and transparency of the payment process.

## Results

### The basic situation and authority coefficient of experts

16 experts were included in this study. The basic situation is shown in Table 2. Sixteen experts participated in the two rounds of Delphi consultation, and the effective response rate of the two rounds of Delphi was 100.0%. According to calculations, the experts in this study are familiar with the mean coefficient Cs = 0.83, and the mean judgment coefficient Ca = 0.93. Combining Ca and Cs, the authoritative coefficient Cr value is 0.88.

**Table 2:** Basic information on experts.

Basic Information	Number (N = 16)	Percentage (%)
**Age (years)**		
30 ~ 39	1	6.25
40 ~ 49	6	37.50
50 ~ 59	7	43.75
≥60	2	12.50
**Education Level**		
Bachelor’s	3	18.75
Master’s	4	25.00
Doctoral	9	56.25
**Primary Professional Field**		
Child & Adolescent Health & MCH	3	18.75
Maternal and Child Health (MCH)	5	31.25
Pediatrics	2	12.50
Child & Adolescent Health	2	12.50
Preventive Medicine	1	6.25
Health Management	2	12.50
Nursing	1	6.25
**Years of Related Work Experience**		
11-20 years	7	43.75
21-29 years	7	43.75
≥30 years	2	12.50

### Establishment of evaluation index system and modification of items

The project team combined with the previous literature review and expert interview information to form 4 first-level indicators, 8 second-level indicators, and 54 third-level indicators, covering 4 types of common diseases of children and adolescents, such as overweight and obesity, myopia, caries and spinal curvature abnormalities, health determinants, health status and related health work.

In the Delphi expert consultation, 13 indicators were deleted and 14 indicators were added according to the experts’ opinion. Detailed modifications are presented in Supplementary Table1, resulting in a final total of 55 third-level indicators.

### Coordination coefficient and concentration

After two rounds of expert consultation, the coordination coefficient of the importance, feasibility and sensitivity of each indicator has been improved. See Table 3. The second round of index content importance coordination coefficient reaches 0.284, the feasibility coordination coefficient reaches 0.185, and the sensitivity coordination coefficient reaches 0.314, which shows that the consultation results are reliable (average *P* < 0.001).

**Table 3:** Coordination Coefficient of Expert Opinions on the Three-Level Indicators for Monitoring Common Diseases and Health determinants among Children and Adolescents in Shanghai.

Three-Level Indicators	Importance			Feasibility			Sensitivity		
	w	χ2	*P*-value	w	χ2	*P*-value	w	χ2	*P*-value
Round 1	0.186	167.2	<0.001	0.117	105.3	<0.001	0.132	111.6	<0.001
Round 2	0.284	261.1	<0.001	0.185	170.2	<0.001	0.314	288.0	<0.001

Tables 4 and 5, along with Supplementary Tables2 and 3, show the key contents of the third-level index entries for the monitoring of common diseases of children and adolescents in Shanghai and their health determinants, as well as the experts’ scores on importance, feasibility and sensitivity.

**Table 4:** Expert Scoring Status for the Three-Level Indicators of Common Diseases and Health determinants Monitoring among Children and Adolescents in Shanghai- Genetic and Early-Life Factors.

First-Level Indicator	Second-Level Indicator	Key Content of Third-Level Indicator	Coefficient of Variation	Importance			Feasibility			Sensitivity		
				Mean ± SD	Median	Full Score Ratio	Mean ± SD	Median	Full Score Ratio	Mean ± SD	Median	Full Score Ratio
Genetic and Early-Life Factors	Genetic Factors	Whether parents are myopic	0.136	4.41 ± 0.59	4.3	0.44	4.44 ± 0.61	4.5	0.50	4.31 ± 0.58	4.0	0.38
		Whether parents are overweight or Obesity	0.177	4.47 ± 0.78	5.0	0.56	4.25 ± 0.75	4.0	0.38	4.25 ± 0.75	4.0	0.38
	Pregnancy Factors	Whether had gestational diabetes	0.179	4.31 ± 0.75	4.3	0.38	4.00 ± 0.68	4.0	0.13	3.84 ± 0.65	4.0	0.06
		Whether had gestational hypertension	0.201	4.25 ± 0.81	4.3	0.38	3.94 ± 0.73	4.0	0.13	3.66 ± 0.72	4.0	0.00
		Whether is of advanced maternal age	0.208	4.13 ± 0.84	4.0	0.31	4.19 ± 0.79	4.0	0.31	3.75 ± 0.81	4.0	0.06
	Infant and Toddler Factors	Duration of exclusive breastfeeding after birth	0.172	4.13 ± 0.67	4.0	0.25	3.94 ± 0.53	4.0	0.06	3.63 ± 0.70	4.0	0.00
		Duration of breastfeeding after birth	0.187	4.19 ± 0.70	4.0	0.31	3.88 ± 0.57	4.0	0.06	3.63 ± 0.78	4.0	0.06
		Gestational week at delivery	0.181	4.25 ± 0.73	4.3	0.38	4.13 ± 0.57	4.0	0.19	3.84 ± 0.82	4.0	0.13
		Rate of nutritional supplement use at 0–6 years	0.179	4.13 ± 0.67	4.0	0.25	3.91 ± 0.62	4.0	0.13	3.78 ± 0.77	4.0	0.13
		Average daily screen time during infancy and toddlerhood	0.209	4.47 ± 0.86	5.0	0.63	4.00 ± 0.87	4.0	0.31	4.06 ± 0.83	4.0	0.31

**Table 5:** Expert Scoring Status for the Three-Level Indicators of Common Diseases and Health determinants Monitoring among Children and Adolescents in Shanghai-Health-Related Behavioral Factors.

First-Level Indicator	Second-Level Indicator	Key Content of Third-Level Indicator	Coefficient of Variation	Importance			Feasibility			Sensitivity		
Mean ± SD	Median	Full Score Ratio	Mean ± SD	Median	Full Score Ratio	Mean ± SD	Median	Full Score Ratio
Health-Related Behavioral Factors	Behavioral Factors	Weekly average frequency of unhealthy food consumption	0.151	4.80 ± 0.39	5.0	0.75	4.19 ± 0.63	4.0	0.31	4.00 ± 0.61	4.0	0.19
		Average daily sedentary time	0.163	4.72 ± 0.56	5.0	0.75	4.28 ± 0.66	4.0	0.38	4.09 ± 0.75	4.0	0.25
		Average daily sleep duration	0.124	4.99 ± 0.05	5.0	0.94	4.53 ± 0.60	5.0	0.56	4.44 ± 0.68	4.8	0.50
		Average daily non-learning screen time	0.142	4.80 ± 0.39	5.0	0.75	4.03 ± 0.62	4.0	0.19	4.41 ± 0.59	4.3	0.44
		Average daily outdoor activity time on school days	0.118	4.99 ± 0.05	5.0	0.94	4.47 ± 0.60	4.8	0.50	4.47 ± 0.60	4.8	0.50
		Average daily outdoor activity time on non-school days	0.147	4.91 ± 0.26	5.0	0.88	4.28 ± 0.66	4.0	0.38	4.34 ± 0.76	4.8	0.50

## Discussion

Based on a life-course perspective and the conceptual framework of adolescent health [17], this study constructed a risk assessment indicator system for four highly prevalent common diseases (myopia, obesity, dental caries, and spinal curvature abnormalities) in children and adolescents aged 7–19 years, using the Delphi method with two rounds of expert consultation. The final system comprises 55 three-level indicators across five dimensions: social determinants, school environment, health services, genetic and early life factors, and health-related behavioral factors. The results of two rounds of expert consultation showed that the questionnaire response rate was 100.0%. The mean familiarity coefficient (Cs), judgment coefficient (Ca) and authority coefficient (Cr) of experts were 0.83, 0.93 and 0.88. Expert agreement improved from round 1 to round 2 and reached statistical significance.

This system is consistent with the ‘common disease-common cause-common prevention’ concept proposed [18], as it seeks to identify common risk factors to inform the development of comprehensive health intervention strategies. In the school environment dimension, new indicators such as “blackboard illuminance uniformity achievement rate [19]” are directly related to optimizing the physical environment for myopia prevention. Uneven lighting is a risk factor for the development of myopia, therefore, improving classroom lighting can significantly reduce the incidence of myopia [20]; From the perspective of behavioral factors, “average daily sleep duration [21] “Outdoor activity duration [22]” and other indicators accurately identify the risk sources of myopia and obesity behavior. Insufficient sleep may disrupt circadian rhythms and impair ocular development [23]; while inadequate outdoor activity reduces energy expenditure, potentially leading to a positive energy balance that is strongly associated with the onset of obesity [24]. Compared with the World Health Organization (WHO) Global Student Health Survey (World Health Organization. Global School-based Student Health Survey; GSHS) [25], this study includes indicators such as the “establishment rate of health service referral mechanism” (such as the referral rate of spinal curvature abnormalities in children and adolescents) and “early life factors” (such as infants and young children the average daily exposure time of electronic screens during the period), thereby addressing a limitation of the GSHS, which primarily focuses on behavioral factors with less emphasis on environmental and health service indicators. Compared with the National Student Physical Health Standards (Revised in 2014) released in 2014, this study integrates the common influencing factors of multiple diseases (such as sedentary behavior associated with obesity [26] and spinal curvature abnormalities [27]), breaking through the limitations of traditional single-disease monitoring.

This study shifts the paradigm from single-disease monitoring to a shared risk factor-based assessment for obesity, myopia, dental caries, and spinal curvature abnormalities in children and adolescents. Including school environment, health-related behavioral factors, and genetic and early-life factors, the indicator system extends the traditional student health monitoring framework. This indicator system provides an evidence base for screening, referral, and prioritization of interventions.

However, this study also has certain limitations. Some indicators (such as “average daily screen time in infancy”)rely on parental recall, making the data susceptible to recall bias. Therefore, future validation using birth cohort studies is necessary. Regional differences have not been fully considered. For example, the feasibility of “winter and summer sports homework assignment rate” is poor in rural areas. In the future, it is necessary to formulate adaptability standards for different regions. The indicator system was developed in the context of Shanghai’s student health monitoring practice and based on a Chinese expert panel; therefore, its applicability to other regions requires further validation. Furthermore, the indicator framework established in this study comprises 55 indicators. While comprehensive, the large number of indicators may compromise practicality and cost-effectiveness in large-scale monitoring. Future efforts should focus on developing a streamlined core indicator set that retains analytical robustness while enhancing feasibility across diverse implementation scenarios. Finally, the index system needs to verify the validity in monitoring practice.

In summary, this study successfully built a risk assessment indicator system for common diseases in children and adolescents through the Delphi expert consultation method. The system covers a total of 55 indicators in five dimensions: social determinants, school environment, health services, genetic and early life factors, and health-related behavioral factors. This index system provides a standardized tool for measuring the level of factors affecting the health of common diseases of children and adolescents. After the credibility is verified, it can be applied to the practical work of relevant health interventions, policy formulation and health management.

## Supporting information

Supplementary Table 1 Details of indicators deleted and added based on expert recommendations during the Delphi consultation process. The deleted indicators are those deemed irrelevant or impractical by experts, while the added indicators are proposed to enhance the comprehensiveness and applicability of the evaluation system.
(DOCX)

Supplementary Table 2 Expert scoring results for the three-level indicators under the “Social Determinants” dimension. The table includes the coefficient of variation, mean ± standard deviation (SD), median, and full score ratio for each indicator’s importance, feasibility, and sensitivity, reflecting expert consensus and evaluation of indicator performance.
(DOCX)

Supplementary Table 3 Expert scoring results for the three-level indicators under the “Health Outcomes” dimension. Similar to Supplementary Table2, this table presents statistical data on the importance, feasibility, and sensitivity of indicators related to disease prevalence and incidence, providing a basis for indicator optimization and validation.
(DOCX)

Supplementary Information Expert scoring raw data are provided in supplementary information file.zip. (ZIP)

## References

[1] Viner RM, Allen NB, Patton GC. Puberty developmental process and health interventions. Child and Adolescent Health and Development. 2017. doi: 10.1596/978-1-4648-0423-6_ch9 30212144

[2] Banatvala N, Akselrod S, Bovet P, Mendis S. The WHO global action plan for the prevention and control of NCDs 2013–2030. 2023. p. 234–9. doi: 10.4324/9781003306689-36

[3] WHO. World report on vision 2019. Geneva: World Health Organization; 2019. https://www.who.int/Publications/i/Item/9789241516570

[4] WHO. Global strategy and action plan on oral health 2023–2030. World Health Organization; 2024. https://www.who.int/publications/i/item/9789240090538

[5] China NHCotPsRo. Five‑Health Promotion Action Plan for Children and Adolescents (2026–2030). National Health Commission of the People’s Republic of China; 2025. https://www.nhc.gov.cn/fys/c100078/202512/9c3da4badfb34026b5fabbf5853105ee.shtml

[6] The Lancet Public Health. Time to tackle obesogenic environments. Lancet Public Health. 2025;10(3):e165. doi: 10.1016/S2468-2667(25)00049-0 40044241

[7] Dong L, Zhou W-D, Hu Y-B, Wei L, Cao W, Chen N-H, et al. Prevalence of myopia and axial length distribution in China: the Wuhu children and adolescents eye study. Invest Ophthalmol Vis Sci. 2025;66(6):33. doi: 10.1167/iovs.66.6.33 40492984 PMC12165258

[8] Zhao L, Jiang X, Zhang W, Hao L, Zhang Y, Wu S, et al. Prevalence and risk factors of myopia among children and adolescents in Hangzhou. Sci Rep. 2024;14(1):24615. doi: 10.1038/s41598-024-73388-7 39426975 PMC11490480

[9] Hao C, Hao Y, Lou X, Wang X, Liu W, Zhou H, et al. Secular trends of dental caries and association with nutritional status: a retrospective analysis of 16,199 Chinese students from three successive national surveys from 2010 to 2019. Front Public Health. 2024;12:1379767. doi: 10.3389/fpubh.2024.1379767 38841684 PMC11150691

[10] Li Y, Xiang Y, Wang Y, Zhang X, Xin Y, Xue H, et al. Detection rate and risk factors of abnormal spinal curvature among children and adolescents - Jiangsu province, China, 2021-2023. China CDC Wkly. 2025;7(6):208–16. doi: 10.46234/ccdcw2025.033 39975940 PMC11832448

[11] Littleton SH, Berkowitz RI, Grant SFA. Genetic determinants of childhood obesity. Mol Diagn Ther. 2020;24(6):653–63. doi: 10.1007/s40291-020-00496-1 33006084 PMC7680380

[12] Poorolajal J, Sahraei F, Mohamdadi Y, Doosti-Irani A, Moradi L. Behavioral factors influencing childhood obesity: a systematic review and meta-analysis. Obes Res Clin Pract. 2020;14(2):109–18. doi: 10.1016/j.orcp.2020.03.002 32199860

[13] Huang Z, Song D, Tian Z, Wang Y, Tian K. Prevalence and associated factors of myopia among adolescents aged 12-15 in Shandong Province, China: a cross-sectional study. Sci Rep. 2024;14(1):17289. doi: 10.1038/s41598-024-68076-5 39068195 PMC11283487

[14] Münte E, Zhang X, Khurana A, Hartmann P. Prevalence of extremely severe obesity and metabolic dysfunction among US Children and adolescents. JAMA Netw Open. 2025;8(7):e2521170. doi: 10.1001/jamanetworkopen.2025.21170 40668581 PMC12268495

[15] Aarestrup J, Andersen EW, Pedersen DC, Bjerregaard LG, Baker JL. Disease patterns across the life course by childhood BMI group. Obesity (Silver Spring). 2026;34(1):237–45. doi: 10.1002/oby.70072 41253727

[16] Marcus C, Danielsson P, Hagman E. Pediatric obesity-Long-term consequences and effect of weight loss. J Intern Med. 2022;292(6):870–91. doi: 10.1111/joim.13547 35883220 PMC9805112

[17] Tao FB. The importance of reconstructing the health assessment indicator system for adolescents. Chinese Journal of School Health. 2014;35(1):1–4. doi: 10.16835/j.cnki.1000-9817.2014.01.001

[18] Dong B, Feng TS, Song Y. Implementing high-quality school health standards to promote joint prevention of common diseases and multimorbidity among students. Zhonghua Yu Fang Yi Xue Za Zhi. 2025;59(2):138–43. doi: 10.3760/cma.j.cn112150-20240826-00683 39748187

[19] Zhonghui L, Ke X, Zhiying S, Xin Z, Baojia F, Gang F. Relationship between classroom lighting and poor vision of students in primary and secondary schools in Tianjin. Chinese Journal of School Health. 2021. doi: 10.16835/j.cnki.1000-9817.2021.08.025

[20] Suh Y-W, Ha S-G, Kim S-H. Effect of classroom illuminance on the development and progression of myopia in school children. Korean J Ophthalmol. 2022;36(3):194–201. doi: 10.3341/kjo.2021.0170 35067020 PMC9194730

[21] Li R, Chen Y, Zhao A, Huang L, Long Z, Kang W, et al. Relationships between sleep duration, timing, consistency, and chronotype with myopia among school-aged children. J Ophthalmol. 2022;2022:7071801. doi: 10.1155/2022/7071801 35903175 PMC9325560

[22] Obregón AM, Pettinelli PP, Santos JL. Childhood obesity and eating behaviour. J Pediatr Endocrinol Metab. 2015;28(5–6):497–502. doi: 10.1515/jpem-2014-0206 25389988

[23] Zhao X, He Y, Zhang J, Lin S, Zou H, Ma Y. Effects of insufficient sleep on myopia in children: a systematic review and meta-analysis. Nat Sci Sleep. 2024;16:1387–406. doi: 10.2147/NSS.S472748 39308665 PMC11416795

[24] Ali A, Al-Ani O, Al-Ani F. Children’s behaviour and childhood obesity. Pediatr Endocrinol Diabetes Metab. 2024;30(3):148–58. doi: 10.5114/pedm.2024.142586 39451187 PMC11538919

[25] Abdalmaleki E, Abdi Z, Isfahani SR, Safarpoor S, Haghdoost B, Sazgarnejad S, et al. Global school-based student health survey: country profiles and survey results in the eastern Mediterranean region countries. BMC Public Health. 2022;22(1):130. doi: 10.1186/s12889-022-12502-8 35045855 PMC8767753

[26] Jebeile H, Kelly AS, O’Malley G, Baur LA. Obesity in children and adolescents: epidemiology, causes, assessment, and management. Lancet Diabetes Endocrinol. 2022;10(5):351–65. doi: 10.1016/S2213-8587(22)00047-X 35248172 PMC9831747

[27] Tu Q, Xu W, Feng Y, Zhang Z, Zhuang W. Prevalence and determinants of adolescent idiopathic scoliosis from school screening in Xiaoshan District, Hangzhou, China. Front Public Health. 2025;13:1595793. doi: 10.3389/fpubh.2025.1595793 40606103 PMC12213709

