# Supplementary material for: A risk assessment indicator system for common diseases in children and adolescents
Source: PLoS One. 2026 Jun 17;21(6):e0351870. doi: 10.1371/journal.pone.0351870 (PMC13274816; doi:10.1371/journal.pone.0351870)
Supplement: Supplementary Table 1 — The deleted indicators are those deemed irrelevant or impractical by experts, while the added indicators are proposed to enhance the comprehensiveness and applicability of the evaluation system. (DOCX) [file pone.0351870.s001.docx]

**Supplementary Table 1: The expert recommended the removal of certain indicators and the addition of new ones.**

| Delete indicators | Increase indicators |
| --- | --- |
| Participation rate of parents in health education activities | Achievement rate of blackboard surface illuminance uniformity |
| Control rate of electronic products in schools | Achievement rate of classroom desktop illuminance uniformity |
| Compliance rate of desk and chair allocation | Achievement rate of classroom noise standards |
| Whether parents have spinal curvature abnormalities | Establishment rate of referral tracking mechanism for common diseases in children and adolescents |
| Whether the mother had excessive weight gain during pregnancy | Average daily electronic screen exposure duration in infancy and toddlerhood |
| Whether cholestasis syndrome occurred during pregnancy | Average daily sedentary time |
| Birth weight | Average daily outdoor activity time on non-school days |
| Participation rate of health education activities for common diseases | Screened high myopia prevalence of children and adolescents at provincial, municipal, and district (county) levels |
| Occurrence rate of sedentary behavior in a week | Overweight incidence of children and adolescents at provincial, municipal, and district (county) levels |
| Calcium intake rate | Obesity incidence of children and adolescents at provincial, municipal, and district (county) levels |
| Achievement rate of standard reading and writing posture | Dental caries incidence of children and adolescents at provincial, municipal, and district (county) levels |
| Vitamin D intake rate | Spinal curvature abnormality incidence of children and adolescents at provincial, municipal, and district (county) levels |
| Spinal curvature abnormalities rate of children and adolescents at provincial, municipal, and district (county) levels | Screened myopia incidence of children and adolescents at provincial, municipal, and district (county) levels |
|  | Screened high myopia incidence of children and adolescents at provincial, municipal, and district (county) levels |
