# Supplementary material for: A risk assessment indicator system for common diseases in children and adolescents
Source: PLoS One. 2026 Jun 17;21(6):e0351870. doi: 10.1371/journal.pone.0351870 (PMC13274816; doi:10.1371/journal.pone.0351870)
Supplement: Supplementary Table 2 — The table includes the coefficient of variation, mean ± standard deviation (SD), median, and full score ratio for each indicator’s importance, feasibility, and sensitivity, reflecting expert consensus and evaluation of indicator performance. (DOCX) [file pone.0351870.s002.docx]

| **Supplementary Table 2: Expert Scoring Status for the Three-Level Indicators of Common Diseases and Health determinants Monitoring among Children and Adolescents in Shanghai-Social Determinants** | | | | | | | | | | | | |
| --- | --- | --- | --- | --- | --- | --- | --- | --- | --- | --- | --- | --- |
| First-Level Indicator | Second-Level Indicator | Key Content of Third-Level Indicator | Coefficient of Variation | Importance | | | Feasibility | | | Sensitivity | | |
|  |  |  |  | Mean ± SD | Median | Full Score Ratio | Mean ± SD | Median | Full Score Ratio | Mean ± SD | Median | Full Score Ratio |
| Social Determinants | Family Environment | Parental attention rate to health information | 0.164 | 4.86 ± 0.33 | 5.0 | 0.81 | 4.31 ± 0.58 | 4.0 | 0.38 | 4.03 ± 0.87 | 4.0 | 0.31 |
|  |  | Parental attention rate to oral health | 0.164 | 4.80 ± 0.39 | 5.0 | 0.75 | 4.44 ± 0.70 | 5.0 | 0.56 | 3.97 ± 0.76 | 4.0 | 0.25 |
|  |  | Parental attention rate to vision health | 0.149 | 4.86 ± 0.33 | 5.0 | 0.81 | 4.50 ± 0.61 | 5.0 | 0.56 | 4.09 ± 0.75 | 4.0 | 0.25 |
|  |  | Parental attention rate to children and adolescents' exercise | 0.164 | 4.92 ± 0.25 | 5.0 | 0.88 | 4.31 ± 0.68 | 4.0 | 0.44 | 4.03 ± 0.80 | 4.0 | 0.25 |
|  |  | Parental attention rate to overweight and obesity | 0.176 | 4.92 ± 0.25 | 5.0 | 0.88 | 4.25 ± 0.83 | 4.5 | 0.50 | 4.16 ± 0.86 | 4.3 | 0.44 |
|  |  | Parental attention rate to spinal curvature abnormalities | 0.215 | 4.59 ± 0.47 | 5.0 | 0.56 | 3.81 ± 0.81 | 4.0 | 0.25 | 3.59 ± 0.89 | 3.0 | 0.19 |
|  |  | Parental attention rate to family dietary health | 0.158 | 4.74 ± 0.43 | 5.0 | 0.69 | 4.31 ± 0.58 | 4.0 | 0.38 | 4.03 ± 0.80 | 4.0 | 0.25 |
|  | School Environment | Implementation rate of common disease prevention and control in schools | 0.139 | 4.80 ± 0.39 | 5.0 | 0.75 | 4.66 ± 0.58 | 5.0 | 0.69 | 4.13 ± 0.67 | 4.0 | 0.25 |
| **Supplementary Table 2 (Continued): Expert Scoring Status for the Three-Level Indicators of Common Diseases and Health determinants Monitoring among Children and Adolescents in Shanghai-Social Determinants** | | | | | | | | | | | | |
| First-Level Indicator | Second-Level Indicator | Key Content of Third-Level Indicator | Coefficient of Variation | Importance | | | Feasibility | | | Sensitivity | | |
|  |  |  |  | Mean ± SD | Median | Full Score Ratio | Mean ± SD | Median | Full Score Ratio | Mean ± SD | Median | Full Score Ratio |
| Social Determinants | School Environment | Achievement rate of students' average daily outdoor activity time at school | 0.138 | 4.93 ± 0.24 | 5.0 | 0.88 | 4.53 ± 0.78 | 5.0 | 0.63 | 4.28 ± 0.56 | 4.0 | 0.31 |
|  |  | Achievement rate of physical education curriculum implementation in schools | 0.139 | 4.86 ± 0.33 | 5.0 | 0.81 | 4.66 ± 0.58 | 5.0 | 0.69 | 4.16 ± 0.70 | 4.0 | 0.31 |
|  |  | Assignment rate of physical education homework during winter and summer vacations | 0.215 | 4.13 ± 0.67 | 4.0 | 0.25 | 4.00 ± 0.77 | 4.0 | 0.25 | 3.31 ± 0.77 | 3.0 | 0.06 |
|  |  | Achievement rate of average blackboard illuminance | 0.149 | 4.53 ± 0.60 | 5.0 | 0.56 | 4.63 ± 0.45 | 5.0 | 0.56 | 3.88 ± 0.60 | 4.0 | 0.13 |
|  |  | Achievement rate of average desktop illuminance | 0.153 | 4.47 ± 0.60 | 4.8 | 0.50 | 4.50 ± 0.47 | 4.5 | 0.44 | 3.81 ± 0.63 | 4.0 | 0.13 |
|  |  | Average completion time of school homework | 0.204 | 4.66 ± 0.46 | 5.0 | 0.63 | 4.09 ± 0.75 | 4.0 | 0.31 | 3.50 ± 0.79 | 3.0 | 0.13 |
|  |  | Formulation rate of nutrient intake and quantitative recipes for cafeteria lunches | 0.172 | 4.50 ± 0.59 | 4.8 | 0.50 | 4.50 ± 0.59 | 4.8 | 0.50 | 3.72 ± 0.71 | 4.0 | 0.06 |
|  |  | Achievement rate of blackboard illuminance uniformity | 0.192 | 4.16 ± 1.00 | 4.0 | 0.38 | 4.28 ± 0.53 | 4.0 | 0.25 | 3.75 ± 0.64 | 4.0 | 0.06 |
| **Supplementary Table 2 (Continued): Expert Scoring Status for the Three-Level Indicators of Common Diseases and Health determinants Monitoring among Children and Adolescents in Shanghai-Social Determinants** | | | | | | | | | | | | |
| First-Level Indicator | Second-Level Indicator | Key Content of Third-Level Indicator | Coefficient of Variation | Importance | | | Feasibility | | | Sensitivity | | |
|  |  |  |  | Mean ± SD | Median | Full Score Ratio | Mean ± SD | Median | Full Score Ratio | Mean ± SD | Median | Full Score Ratio |
| Social Determinants | School Environment | Achievement rate of desktop illuminance uniformity | 0.183 | 4.41 ± 0.59 | 4.3 | 0.44 | 4.22 ± 0.71 | 4.0 | 0.25 | 3.69 ± 0.75 | 4.0 | 0.06 |
|  |  | Achievement rate of classroom noise standards | 0.168 | 4.16 ± 0.76 | 4.3 | 0.31 | 4.16 ± 0.61 | 4.0 | 0.25 | 3.75 ± 0.56 | 4.0 | 0.06 |
|  | Health Services | Organization rate of student physical examinations in schools | 0.142 | 4.74 ± 0.56 | 5.0 | 0.75 | 4.93 ± 0.24 | 5.0 | 0.88 | 3.88 ± 0.48 | 4.0 | 0.06 |
|  |  | Coverage rate of school physical examinations | 0.118 | 4.93 ± 0.24 | 5.0 | 0.88 | 4.99 ± 0.05 | 5.0 | 0.94 | 4.03 ± 0.51 | 4.0 | 0.13 |
|  |  | Timely feedback rate of physical examination results | 0.143 | 4.61 ± 0.48 | 5.0 | 0.56 | 4.55 ± 0.70 | 5.0 | 0.63 | 4.16 ± 0.61 | 4.0 | 0.25 |
|  |  | Establishment rate of student health management records in schools | 0.134 | 4.64 ± 0.45 | 5.0 | 0.56 | 4.52 ± 0.47 | 4.7 | 0.44 | 3.94 ± 0.56 | 4.0 | 0.13 |
|  |  | Referral rate for spinal curvature abnormalities among children and adolescents | 0.150 | 4.80 ± 0.39 | 5.0 | 0.75 | 4.16 ± 0.70 | 4.0 | 0.31 | 4.13 ± 0.60 | 4.0 | 0.25 |
|  |  | Referral rate for myopia among children and adolescents | 0.133 | 4.86 ± 0.33 | 5.0 | 0.81 | 4.41 ± 0.69 | 4.8 | 0.50 | 4.28 ± 0.56 | 4.0 | 0.31 |
| **Supplementary Table 2 (Continued): Expert Scoring Status for the Three-Level Indicators of Common Diseases and Health determinants Monitoring among Children and Adolescents in Shanghai-Social Determinants** | | | | | | | | | | | | |
| First-Level Indicator | Second-Level Indicator | Key Content of Third-Level Indicator | Coefficient of Variation | Importance | | | Feasibility | | | Sensitivity | | |
|  |  |  |  | Mean ± SD | Median | Full Score Ratio | Mean ± SD | Median | Full Score Ratio | Mean ± SD | Median | Full Score Ratio |
| Social Determinants | Health Services | Referral rate for overweight and obesity among children and adolescents | 0.150 | 4.86 ± 0.33 | 5.0 | 0.81 | 4.16 ± 0.70 | 4.0 | 0.31 | 4.09 ± 0.57 | 4.0 | 0.19 |
|  |  | Referral rate for dental caries among children and adolescents | 0.149 | 4.80 ± 0.39 | 5.0 | 0.75 | 4.28 ± 0.75 | 4.3 | 0.44 | 4.19 ± 0.61 | 4.0 | 0.25 |
|  |  | Establishment rate of referral tracking mechanism for common diseases among children and adolescents | 0.214 | 4.55 ± 0.70 | 5.0 | 0.63 | 4.03 ± 0.94 | 4.0 | 0.38 | 3.88 ± 0.86 | 4.0 | 0.25 |
