# Supplementary material for: A risk assessment indicator system for common diseases in children and adolescents
Source: PLoS One. 2026 Jun 17;21(6):e0351870. doi: 10.1371/journal.pone.0351870 (PMC13274816; doi:10.1371/journal.pone.0351870)
Supplement: Supplementary Table 3 — Similar to Supplementary Table2, this table presents statistical data on the importance, feasibility, and sensitivity of indicators related to disease prevalence and incidence, providing a basis for indicator optimization and validation. (DOCX) [file pone.0351870.s003.docx]

| **Supplementary Table 3: Expert Scoring Status for the Three-Level Indicators of Common Diseases and Health determinants Monitoring among Children and Adolescents in Shanghai-Health Outcomes** | | | | | | | | | | | | |
| --- | --- | --- | --- | --- | --- | --- | --- | --- | --- | --- | --- | --- |
| First-Level Indicator | Second-Level Indicator | Key Content of Third-Level Indicator | Coefficient of Variation | Importance | | | Feasibility | | | Sensitivity | | |
|  |  |  |  | Mean ± SD | Median | Full Score Ratio | Mean ± SD | Median | Full Score Ratio | Mean ± SD | Median | Full Score Ratio |
| Health Outcomes | Disease Prevalence | Prevalence of overweight among children and adolescents | 0.103 | 4.99 ± 0.05 | 5.0 | 0.94 | 4.66 ± 0.46 | 5.0 | 0.63 | 4.47 ± 0.60 | 4.8 | 0.50 |
|  |  | Prevalence of obesity among children and adolescents | 0.091 | 4.99 ± 0.05 | 5.0 | 0.94 | 4.66 ± 0.46 | 5.0 | 0.63 | 4.53 ± 0.48 | 4.8 | 0.50 |
|  |  | Prevalence of dental caries among children and adolescents | 0.108 | 4.93 ± 0.24 | 5.0 | 0.88 | 4.66 ± 0.46 | 5.0 | 0.63 | 4.41 ± 0.59 | 4.3 | 0.44 |
|  |  | Prevalence of spinal curvature abnormalities among children and adolescents | 0.140 | 4.86 ± 0.33 | 5.0 | 0.81 | 4.41 ± 0.69 | 4.8 | 0.50 | 4.28 ± 0.66 | 4.0 | 0.38 |
|  |  | Prevalence of screened myopia among children and adolescents | 0.088 | 4.99 ± 0.05 | 5.0 | 0.94 | 4.72 ± 0.43 | 5.0 | 0.69 | 4.53 ± 0.48 | 4.8 | 0.50 |
|  |  | Prevalence of screened high myopia among children and adolescents | 0.131 | 4.80 ± 0.39 | 5.0 | 0.75 | 4.34 ± 0.68 | 4.3 | 0.44 | 4.53 ± 0.60 | 5.0 | 0.56 |
|  |  | Incidence of overweight among children and adolescents | 0.148 | 4.68 ± 0.58 | 5.0 | 0.69 | 4.41 ± 0.59 | 4.3 | 0.44 | 4.41 ± 0.77 | 4.8 | 0.50 |
|  |  | \| Incidence of obesity among children and adolescents \| \| --- \| | 0.106 | 4.68 ± 0.46 | 5.0 | 0.63 | 4.47 ± 0.48 | 4.3 | 0.44 | 4.53 ± 0.48 | 4.8 | 0.50 |
|  |  | \| Incidence of dental caries among children and adolescents \| \| --- \| | 0.149 | 4.61 ± 0.59 | 5.0 | 0.63 | 4.34 ± 0.58 | 4.0 | 0.38 | 4.41 ± 0.77 | 4.8 | 0.50 |
|  |  | Incidence of spinal curvature abnormalities among children and adolescents | 0.176 | 4.61 ± 0.59 | 5.0 | 0.63 | 4.09 ± 0.75 | 4.0 | 0.25 | 4.22 ± 0.81 | 4.0 | 0.38 |
|  |  | Incidence of screened myopia among children and adolescents | 0.103 | 4.74 ± 0.43 | 5.0 | 0.69 | 4.47 ± 0.48 | 4.3 | 0.44 | 4.59 ± 0.47 | 5.0 | 0.56 |
|  |  | Incidence of screened high myopia among children and adolescents | 0.134 | 4.68 ± 0.46 | 5.0 | 0.63 | 4.22 ± 0.64 | 4.0 | 0.31 | 4.53 ± 0.60 | 5.0 | 0.56 |
