# Supplementary material for: A risk assessment indicator system for common diseases in children and adolescents
Source: PLoS One. 2026 Jun 17;21(6):e0351870. doi: 10.1371/journal.pone.0351870 (PMC13274816; doi:10.1371/journal.pone.0351870)
Supplement: Attachment — Submitted filename: Response to Reviewers.docx [file pone.0351870.s007.docx]

Response to Reviewers

Dear Editors and Reviewers,

We would like to express our sincere gratitude to you for your valuable time, meticulous review, and constructive comments on our manuscript entitled “A Risk Assessment Indicator System for Common Diseases in Children and Adolescents”. These opinions help to improve academic rigor of our article Based on your suggestion and request,we have made corrected modifications on the revised manuscript. We have carefully considered all the suggestions and made corresponding revisions to improve the quality and rigor of the manuscript. Below is a detailed response to each comment.

1. Please ensure that your manuscript meets PLOS ONE's style requirements, including those for file naming. The PLOS ONE style templates can be found at https://journals.plos.org/plosone/s/file?id=wjVg/PLOSOne_formatting_sample_main_body.pdf and https://journals.plos.org/plosone/s/file?id=ba62/PLOSOne_formatting_sample_title_authors_affiliations.pdf

- We have carefully reviewed the PLOS ONE style templates and adjusted the manuscript to fully meet the journal’s formatting requirements, including file naming conventions. All sections, headings, and citations have been standardized in accordance with the provided guidelines.

2. Please provide additional details regarding participant consent. In the ethics statement in the Methods and online submission information, please ensure that you have specified (1) whether consent was informed and (2) what type you obtained (for instance, written or verbal, and if verbal, how it was documented and witnessed). If your study included minors, state whether you obtained consent from parents or guardians. If the need for consent was waived by the ethics committee, please include this information.

- In the Ethics Statement section of the Methods, we have supplemented the following details regarding participant consent:
- Oral informed consent was obtained from all experts prior to their engagement in the consultation.
- The first page of the consultation questionnaire included a formal invitation letter that clearly specified the research objectives, scope of the consultation, and other relevant information. Questionnaires were distributed via email, and completed responses were returned by the same means.
- This study employs the Delphi technique for expert consultation with 16 experts. This study did not involve minors. Thus, consent from parents or guardians was not applicable.

3. We note that your Data Availability Statement is currently as follows: All relevant data are within the manuscript and its Supporting Information files.

Please confirm at this time whether or not your submission contains all raw data required to replicate the results of your study.

- The original data were not included in the initial submission, but they are complete and available.We will upload the original data as supplementary information file.

4. Thank you for stating the following financial disclosure:

Three-year Action Plan for Strengthening the Construction of Public Health System in Shanghai（2023—2025）（GWVI-11.1-32）

Please state what role the funders took in the study. If the funders had no role, please state: "The funders had no role in study design, data collection and analysis, decision to publish, or preparation of the manuscript."

If this statement is not correct you must amend it as needed.

Please include this amended Role of Funder statement in your cover letter; we will change the online submission form on your behalf.

- We have included the Role of Funder statement in the cover letter, with the specific wording as follows: “The funders had no role in study design, data collection and analysis, decision to publish, or preparation of the manuscript.” This revision fully addresses the editor’s requirement.

5. We notice that your supplementary [tables 1, 2 and 3] are included in the manuscript file. Please remove them and upload them with the file type 'Supporting Information'. Please ensure that each Supporting Information file has a legend listed in the manuscript after the references list.

- We have prepared corresponding legends for each Supporting Information file (Supplementary Tables 1–3) and included them in the manuscript after the references list as required.

**Reviewer #1:**

Abstract

• Start each subsection on a new line.

• Add a brief introductory background before the objectives.

• Strengthen the justification briefly.

- We sincerely appreciate your constructive suggestion. We have revised the abstract to start each subsection (Background, Objective, Methods, Results, Conclusion) on a new line.
- A brief introductory background has been added before the objectives to clarify the significance of constructing the indicator system. The background content is as follows: “Adolescence is a critical stage for healthy development. Currently, common diseases such as myopia, obesity, dental caries, and spinal curvature abnormalities are highly prevalent among children and adolescents, with an obvious trend of younger onset. These diseases are caused by the complex interaction of multi-dimensional factors. However, existing interventions are mostly limited to individual diseases, lacking systematic prevention and control tools. Therefore, there is an urgent need to construct a scientific evaluation index system for health-influencing factors.”
- The justification for the study has been strengthened by briefly mentioning the policy, and the revision is as follows: “Guided by China's Outline for Children's Development (2021-2030) and the "Healthy China 2030" Planning Outline, a three-level indicator framework was initially formulated through literature research and expert meetings.”

Introduction

• Please check and standardize the referencing style throughout the manuscript.

• Lines 26: The statement that obesity, myopia, dental caries, and spinal curvature abnormalities are described as “common” requires justification. Please provide prevalence data and improve the flow of supporting evidence.

• Please clarify whether communicable diseases are being considered. If not, specify that the focus is on common Non-Communicable Diseases (NCDs).

• Lines 38: Statements on the immediate and long-term health consequences of childhood and adolescent obesity should be supported with appropriate references and relevant statistics.

• Lines 44: Please explain how the common diseases were identified and selected, and clearly state which diseases were included.

- Thank you for your valuable suggestion. We have thoroughly checked and standardized the referencing style throughout the manuscript to adhere to PLOS ONE’s requirements, including consistent formatting of author names, journal titles, and DOI inclusion where available.
- Obesity, myopia, dental caries, and spinal curvature abnormalities are explicitly classified as “common diseases” in relevant Chinese government policy documents. To strengthen our argument, we supplemented the corresponding prevalence data. The revised content is as follows: “Accordingly, National Health Commission of the People’s Republic of China has formulated the "Five‑Health" Promotion Action Plan for Children and Adolescents (2026–2030)[1], which identifies obesity, myopia, dental caries, and spinal curvature abnormalities as key priority areas for prevention and control. From the perspective of specific data, according to WHO data in 2022, 390 million children and adolescents aged 5–19 years had overweight, including 160 million with obesity[7]”
- We have stated that this study focuses on common non-communicable diseases (NCDs) in children and adolescents explicitly identified in relevant Chinese government policy documents. The revised content is as follows: “The World Health Organization (WHO) has included childhood myopia, dental caries, and obesity in its non‑communicable disease prevention and control framework[2]. Accordingly, National Health Commission of the People’s Republic of China has formulated the "Five‑Health" Promotion Action Plan for Children and Adolescents (2026–2030)[1], which identifies obesity, myopia, dental caries, and spinal curvature abnormalities as key priority areas for prevention and control.”
- For the immediate and long-term health consequences of childhood and adolescent obesity, we have added relevant references and statistics. The revised content is as follows: “For example, a large cross-sectional study based on nationally representative data of U.S. children and adolescents showed that, compared with those with mild-to-moderate obesity (body mass index [BMI] ≥ 95th percentile to <160% of the 95th percentile), individuals with extremely severe obesity (BMI ≥160% of the 95th percentile) had a 4.94-fold higher risk of prediabetes/diabetes and a 6.74-fold higher risk of metabolic dysfunction–associated steatotic liver disease[14]. A large life-course cohort study[15] has provided compelling evidence for the long-term health impacts of childhood obesity. Compared with children whose childhood BMI was between the 15th and 50th percentiles, those in the obese group (≥99th percentile) had hazard ratios (HRs) of 2.00 and 1.68 for developing endocrine/metabolic diseases and circulatory system diseases in adulthood.”
- The selection of common diseases in this study is primarily based on the explicit definition in China’s "Five-Health" Promotion Action Plan for Children and Adolescents (2026–2030) — this policy classifies myopia, obesity, dental caries, and spinal curvature abnormalities as common diseases requiring priority prevention and control among children and adolescents, hence this study focuses on these four conditions with clear policy orientation.

Methods

• The Ethics section should be placed as the final subsection of the Methods.

• Lines 79: Please describe how the first-, second-, and third-level indicators were developed, including the rationale for classification and the process used to select experts.

- We are grateful for your professional suggestion. We have relocated the Ethics Statement to the final subsection of the Methods section, as requested.
- We have detailed the development process of first-, second-, and third-level indicators, as well as the expert selection rationale. The revised content is as follows: “
- Subjects

Delphi experts were selected based on the following criteria : (1) Professional Field: Experts should have rich practical experience in fields such as Child & Adolescent Health, Maternal and Child Health (MCH), Pediatrics, Preventive Medicine, Health Management, and Nursing. (2) Academic Level: They should possess a relatively high academic level in relevant fields and have a certain professional influence in the industry. (3) Title and Qualifications: They need to hold an associate senior or above professional title, or have an equivalent management rank. (4) Work Experience: They should have at least 11 years of work experience in related fields, with solid industry accumulation and practical knowledge. Potential experts were initially identified through recommendations from collaborative institutions and peers. After eligibility verification, consultation questionnaires were sent via email. Ultimately, a total of 16 experts from Centers for Disease Control and Prevention, universities, hospitals, maternal and child health centers, health commissions, primary/secondary schools, and community health service centers were enrolled in this study.

- Method Preliminary index system

The first-level and second-level indicators were classified based on national policies and also with reference to the life course perspective-based adolescent health assessment framework proposed by Tao FB (2014)[17]. Guided by core policy documents including the National Program for Child Development (2021-2030) and the Healthy China 2030 Plan Outline, this study integrated the life course perspective and adolescent health conceptual framework to divide the content of children and adolescents' health monitoring into 4 first-level indicators and 8 second-level indicators. The construction of third-level indicators was mainly based on literature review, existing monitoring systems, and the needs of expert consensus. A systematic search was conducted across Chinese and English databases (e.g., CNKI, Wanfang Data, PubMed) and official government websites, using keywords such as "children", "adolescents", "students", "myopia", "overweight", "obesity", "dental caries", "spinal curvature abnormalities", "Shanghai", "intervention", "management", "evaluation", "Myopia", "Students", "Adolescents", "Children", "Oral", "Scoliosis", "Caries", "Shanghai", and "Obesity". By incorporating mature indicators from the Shanghai Student Common Diseases and Health Influencing Factors Monitoring and Intervention Scale, a total of 54 third-level indicators were finally formulated.

- Form a pool of index items

Building on these preliminary efforts, the research team integrated policy requirements, theoretical frameworks, literature evidence, and existing monitoring indicators to establish an evaluation indicator inventory comprising 4 first-level indicators, 8 second-level indicators, and 54 third-level indicators. This inventory covers health determinants, health status, and relevant health work related to overweight and obesity, myopia, dental caries, and spinal curvature abnormalities in children and adolescents. Based on this repository, an expert consultation questionnaire was designed for subsequent Delphi method implementation or expert consultation procedures.”

Discussion

• Add a brief summary of the key findings at the beginning of the Discussion section.

References

• Please include DOIs where available and ensure that all references adhere strictly to the journal’s formatting style.

- We are grateful for your professional comment. We have added a brief summary of key findings at the beginning of the Discussion section. The revised statement reads as follows: “Based on a life-course perspective and the conceptual framework of adolescent health[18], this study constructed a risk assessment indicator system for four highly prevalent common diseases (myopia, obesity, dental caries, and spinal curvature abnormalities) in children and adolescents aged 7–19 years, using the Delphi method with two rounds of expert consultation. The final system comprises 55 three-level indicators across five dimensions: social determinants, school environment, health services, genetic and early life factors, and health-related behavioral factors.”
- We have supplemented DOIs for all applicable references and standardized the formatting to comply with PLOS ONE’s reference style. Some Chinese core journal articles do not have an official DOI.

Reviewer #2: The authors have presented a detailed report on their study to develop a risk assessment indicator system for common diseases in children and adolescents. I have some suggestions :

1. Line 8 : Please make the objective more detailed eg which common diseases have been focused on, here? What are the ages of the subjects under study? What was the overall goal of the study?

- We are grateful for your professional comment. We have made the objective more detailed by clarifying the target diseases, subject age range, and overall goal. The revised statement reads as follows: “This study focuses on children and adolescents aged 7-19 years, with a specific emphasis on four highly prevalent common diseases: myopia, obesity, dental caries, and spinal curvature abnormalities. It aims to construct a three-level evaluation indicator system covering multi-dimensional health-influencing factors, providing a standardized and operable evaluation tool for the comprehensive prevention and control of common diseases in children and adolescents as well as the formulation of relevant policies, ultimately improving the health status of the child and adolescent population.”

Once again, we would like to thank the Editors and Reviewers for their valuable comments and suggestions. We believe these revisions have significantly improved the quality and clarity of the manuscript. We hope the revised version meets the publication standards of PLOS ONE and look forward to your further feedback.
