# Supplementary material for: A risk assessment indicator system for common diseases in children and adolescents
Source: PLoS One. 2026 Jun 17;21(6):e0351870. doi: 10.1371/journal.pone.0351870 (PMC13274816; doi:10.1371/journal.pone.0351870)
Supplement: Attachment — Submitted filename: Plosone comment.docx [file pone.0351870.s006.docx]

The authors have presented a detailed report on their study to develop a risk assessment indicator system for common diseases in children and adolescents. It is indeed a very novel approach to improve the health outcomes. I have a few suggestions that could help in the betterment of the manuscript.

1. The Objective section of the Abstract is too long. The authors need to make the objective section of the manuscript briefer.
2. The authors must explain clearly and properly how the common diseases were identified and selected.
3. Line 40 - The term “Keywords” must be written properly.
4. Line 50 - There are multiple syntax errors in the manuscript. The authors must reframe the manuscript by reducing the syntax errors.
5. The authors must also try to mention the prevalence and incidence rates of all the most common diseases in the Introduction section of the manuscript.
6. Some authors must also clearly describe how the ethnic differences between the subjects in the manuscript are so that the race-specificities, age-specificities, or sex-specificities can be clearly depicted in the manuscript.
7. It is suggested that the authors elaborate on the key results in the Discussion section of the manuscript.
8. Multiple references do not have a DOI. Kindly add DOIs to all references in the manuscript, including references 6, 8, 17, 18, 19, 20, and 21.
9. The authors are requested to maintain the same reference style throughout the manuscript.
