# Supplementary material for: A risk assessment indicator system for common diseases in children and adolescents
Source: PLoS One. 2026 Jun 17;21(6):e0351870. doi: 10.1371/journal.pone.0351870 (PMC13274816; doi:10.1371/journal.pone.0351870)
Supplement: Attachment — Submitted filename: Response_to_Reviewers_auresp_2.docx [file pone.0351870.s008.docx]

Response to Reviewers

Dear Editors and Reviewers,

We would like to express our sincere gratitude to you for your valuable time, meticulous review, and constructive comments on our manuscript entitled “A Risk Assessment Indicator System for Common Diseases in Children and Adolescents”. These opinions help to improve academic rigor of our article Based on your suggestion and request,we have made corrected modifications on the revised manuscript. We have carefully considered all the suggestions and made corresponding revisions to improve the quality and rigor of the manuscript. Below is a detailed response to each comment.

1. The Objective section of the Abstract is too long. The authors need to make the objective section of the manuscript briefer.

Thank you very much for your helpful suggestion. We have condensed and streamlined the objective section of the abstract, removing redundant descriptions and keeping only core research purposes. The revised statement reads as follows: Targeting the four key common diseases emphasized in China’s national health policies - myopia, obesity, dental caries, and spinal curvature abnormalities - among children and adolescents aged 7 - 19 years, this study constructed a multi-dimensional risk assessment indicator system to support evidence-based prevention and policy-making for this population.

2. The authors must explain clearly and properly how the common diseases were identified and selected.

Thank you very much for your helpful suggestion. We explain in the Introduction that the four common diseases (myopia, obesity, dental caries, and spinal curvature abnormalities) were selected because they are the key priority diseases identified in China’s national child and adolescent health policies, including the “Five-Health” Promotion Action Plan for Children and Adolescents (2026 – 2030). These diseases pose a serious health burden and have been emphasized by the World Health Organization, so they were selected as the core diseases for this system.

3. Line 40 - The term “Keywords” must be written properly.

Thank you for pointing out this mistake. We have properly revised the writing form of "Keywords" as required.

4. Line 50 - There are multiple syntax errors in the manuscript. The authors must reframe the manuscript by reducing the syntax errors.

We highly appreciate your careful review. We have thoroughly checked and revised the full manuscript, rephrased inappropriate expressions, and corrected existing syntax errors to ensure fluent and standard academic writing.

5. The authors must also try to mention the prevalence and incidence rates of all the most common diseases in the Introduction section of the manuscript.

We fully agree with this suggestion. Relevant data on spinal curvature abnormalities have been supplemented in the introduction section. The revised statement reads as follows: A provincial surveillance study in Jiangsu, China reported an overall detection rate of 2.1% for spinal curvature abnormalities among children and adolescents during 2021 - 2023, highlighting this condition as an important public health issue.

6. Some authors must also clearly describe how the ethnic differences between the subjects in the manuscript are so that the race-specificities, age-specificities, or sex-specificities can be clearly depicted in the manuscript.

Thank you very much for your helpful suggestion. All participants in our study are Chinese without ethnic differences.

7. It is suggested that the authors elaborate on the key results in the Discussion section of the manuscript.

Thank you for your constructive suggestion. We have elaborated on the key results in the Discussion section. The revised statement reads as follows:

- Based on a life-course perspective and the conceptual framework of adolescent health[18], this study constructed a risk assessment indicator system for four highly prevalent common diseases (myopia, obesity, dental caries, and spinal curvature abnormalities) in children and adolescents aged 7–19 years, using the Delphi method with two rounds of expert consultation. The final system comprises 55 three-level indicators across five dimensions: social determinants, school environment, health services, genetic and early life factors, and health-related behavioral factors. The results of two rounds of expert consultation showed that the questionnaire response rate was 100.0%. The mean familiarity coefficient (Cs), judgment coefficient (Ca) and authority coefficient (Cr) of experts were 0.83, 0.93 and 0.88. Expert agreement improved from round 1 to round 2 and reached statistical significance.
- This study shifts the paradigm from single-disease monitoring to a shared risk factor-based assessment for obesity, myopia, dental caries, and spinal curvature abnormalities in children and adolescents. Including school environment, health-related behavioral factors, and genetic and early-life factors, the indicator system extends the traditional student health monitoring framework. This indicator system provides an evidence base for screening, referral, and prioritization of interventions.
- However, this study also has certain limitations. Some indicators (such as "average daily screen time in infancy")rely on parental recall, making the data susceptible to recall bias. Therefore, future validation using birth cohort studies is necessary. Regional differences have not been fully considered. For example, the feasibility of "winter and summer sports homework assignment rate" is poor in rural areas. In the future, it is necessary to formulate adaptability standards for different regions. The indicator system was developed in the context of Shanghai’s student health monitoring practice and based on a Chinese expert panel; therefore, its applicability to other regions requires further validation. Furthermore, the indicator framework established in this study comprises 55 indicators. While comprehensive, the large number of indicators may compromise practicality and cost-effectiveness in large-scale monitoring. Future efforts should focus on developing a streamlined core indicator set that retains analytical robustness while enhancing feasibility across diverse implementation scenarios. Finally, the index system needs to verify the validity in monitoring practice.

8. Multiple references do not have a DOI. Kindly add DOIs to all references in the manuscript, including references 6, 8, 17, 18, 19, 20, and 21.

Thank you for pointing this out. We have added the missing DOIs to all references.

9. The authors are requested to maintain the same reference style throughout the manuscript.

Thank you very much for your helpful suggestion. We have unified and standardized the citation format of all references in manuscript, ensuring consistent reference style throughout the paper.
