# Supplementary material for: A risk assessment indicator system for common diseases in children and adolescents
Source: PLoS One. 2026 Jun 17;21(6):e0351870. doi: 10.1371/journal.pone.0351870 (PMC13274816; doi:10.1371/journal.pone.0351870)
Supplement: Peer review history

# Peer review: Decision Letter 0

**DOI:** 10.1371/journal.pone.0351870.r001

**Authors:** Edison Mworozi

6 Jan 2026

PONE-D-25-58634

A Risk Assessment Indicator System for Common Diseases in Children and Adolescents

PLOS One

Dear Dr. Liang,

Thank you for submitting your manuscript to PLOS ONE. After careful consideration, we feel that it has merit but does not fully meet PLOS ONE’s publication criteria as it currently stands. Therefore, we invite you to submit a revised version of the manuscript that addresses the points raised during the review process.

Please submit your revised manuscript by Feb 20 2026 11:59PM. If you will need more time than this to complete your revisions, please reply to this message or contact the journal office at plosone@plos.org. When you're ready to submit your revision, log on to https://www.editorialmanager.com/pone/ and select the 'Submissions Needing Revision' folder to locate your manuscript file.

Please include the following items when submitting your revised manuscript:

- A letter that responds to each point raised by the academic editor and reviewer(s). You should upload this letter as a separate file labeled 'Response to Reviewers'.
- A marked-up copy of your manuscript that highlights changes made to the original version. You should upload this as a separate file labeled 'Revised Manuscript with Track Changes'.
- An unmarked version of your revised paper without tracked changes. You should upload this as a separate file labeled 'Manuscript'.

If you would like to make changes to your financial disclosure, please include your updated statement in your cover letter. Guidelines for resubmitting your figure files are available below the reviewer comments at the end of this letter.

If applicable, we recommend that you deposit your laboratory protocols in protocols.io to enhance the reproducibility of your results. Protocols.io assigns your protocol its own identifier (DOI) so that it can be cited independently in the future. For instructions see: https://journals.plos.org/plosone/s/submission-guidelines#loc-laboratory-protocols. Additionally, PLOS ONE offers an option for publishing peer-reviewed Lab Protocol articles, which describe protocols hosted on protocols.io. Read more information on sharing protocols at https://plos.org/protocols?utm_medium=editorial-email&utm_source=authorletters&utm_campaign=protocols.

We look forward to receiving your revised manuscript.

Kind regards,

Edison Arwanire Mworozi, M.D

Academic Editor

PLOS One

Journal requirements:

When submitting your revision, we need you to address these additional requirements.

1. Please ensure that your manuscript meets PLOS ONE's style requirements, including those for file naming. The PLOS ONE style templates can be found at https://journals.plos.org/plosone/s/file?id=wjVg/PLOSOne_formatting_sample_main_body.pdf and https://journals.plos.org/plosone/s/file?id=ba62/PLOSOne_formatting_sample_title_authors_affiliations.pdf

2. Please provide additional details regarding participant consent. In the ethics statement in the Methods and online submission information, please ensure that you have specified (1) whether consent was informed and (2) what type you obtained (for instance, written or verbal, and if verbal, how it was documented and witnessed). If your study included minors, state whether you obtained consent from parents or guardians. If the need for consent was waived by the ethics committee, please include this information.

3. We note that your Data Availability Statement is currently as follows: All relevant data are within the manuscript and its Supporting Information files.

Please confirm at this time whether or not your submission contains all raw data required to replicate the results of your study. Authors must share the “minimal data set” for their submission. PLOS defines the minimal data set to consist of the data required to replicate all study findings reported in the article, as well as related metadata and methods (https://journals.plos.org/plosone/s/data-availability#loc-minimal-data-set-definition).

For example, authors should submit the following data:

- The values behind the means, standard deviations and other measures reported;

- The values used to build graphs;

- The points extracted from images for analysis.

Authors do not need to submit their entire data set if only a portion of the data was used in the reported study.

If your submission does not contain these data, please either upload them as Supporting Information files or deposit them to a stable, public repository and provide us with the relevant URLs, DOIs, or accession numbers. For a list of recommended repositories, please see https://journals.plos.org/plosone/s/recommended-repositories.

If there are ethical or legal restrictions on sharing a de-identified data set, please explain them in detail (e.g., data contain potentially sensitive information, data are owned by a third-party organization, etc.) and who has imposed them (e.g., an ethics committee). Please also provide contact information for a data access committee, ethics committee, or other institutional body to which data requests may be sent. If data are owned by a third party, please indicate how others may request data access.

4. Thank you for stating the following financial disclosure:

Three-year Action Plan for Strengthening the Construction of Public Health System in Shanghai（2023—2025）（GWVI-11.1-32）

Please state what role the funders took in the study. If the funders had no role, please state: "The funders had no role in study design, data collection and analysis, decision to publish, or preparation of the manuscript."

If this statement is not correct you must amend it as needed.

Please include this amended Role of Funder statement in your cover letter; we will change the online submission form on your behalf.

5. We notice that your supplementary [tables 1, 2 and 3] are included in the manuscript file. Please remove them and upload them with the file type 'Supporting Information'. Please ensure that each Supporting Information file has a legend listed in the manuscript after the references list.

Additional Editor Comments:

This manuscript requires a major revision. Please address the comments in a robust and timely manner.

Reviewers' comments:

Reviewer's Responses to Questions

**Comments to the Author**

1. Is the manuscript technically sound, and do the data support the conclusions?

The manuscript must describe a technically sound piece of scientific research with data that supports the conclusions. Experiments must have been conducted rigorously, with appropriate controls, replication, and sample sizes. The conclusions must be drawn appropriately based on the data presented.

Reviewer #1: Partly

Reviewer #2: Yes

**********

2. Has the statistical analysis been performed appropriately and rigorously?

Reviewer #1: Yes

Reviewer #2: I Don't Know

**********

3. Have the authors made all data underlying the findings in their manuscript fully available?

The PLOS Data policy requires authors to make all data underlying the findings described in their manuscript fully available without restriction, with rare exception (please refer to the Data Availability Statement in the manuscript PDF file). The data should be provided as part of the manuscript or its supporting information, or deposited to a public repository. For example, in addition to summary statistics, the data points behind means, medians and variance measures should be available. If there are restrictions on publicly sharing data—e.g. participant privacy or use of data from a third party—those must be specified.

Reviewer #1: No

Reviewer #2: Yes

**********

4. Is the manuscript presented in an intelligible fashion and written in standard English?

PLOS ONE does not copyedit accepted manuscripts, so the language in submitted articles must be clear, correct, and unambiguous. Any typographical or grammatical errors should be corrected at revision, so please note any specific errors here.

Reviewer #1: Yes

Reviewer #2: Yes

**********

5. Review Comments to the Author

Please use the space provided to explain your answers to the questions above. You may also include additional comments for the author, including concerns about dual publication, research ethics, or publication ethics. (Please upload your review as an attachment if it exceeds 20,000 characters)

Reviewer #1: Thank you for selecting this important topic and for the detailed work presented. Please address the following comments to further improve the manuscript:

Abstract

• Start each subsection on a new line.

• Add a brief introductory background before the objectives.

• Strengthen the justification briefly.

Introduction

• Please check and standardize the referencing style throughout the manuscript.

• Lines 26: The statement that obesity, myopia, dental caries, and spinal curvature abnormalities are described as “common” requires justification. Please provide prevalence data and improve the flow of supporting evidence.

• Please clarify whether communicable diseases are being considered. If not, specify that the focus is on common Non-Communicable Diseases (NCDs).

• Lines 38: Statements on the immediate and long-term health consequences of childhood and adolescent obesity should be supported with appropriate references and relevant statistics.

• Lines 44: Please explain how the common diseases were identified and selected, and clearly state which diseases were included.

Methods

• The Ethics section should be placed as the final subsection of the Methods.

• Lines 79: Please describe how the first-, second-, and third-level indicators were developed, including the rationale for classification and the process used to select experts.

Discussion

• Add a brief summary of the key findings at the beginning of the Discussion section.

References

• Please include DOIs where available and ensure that all references adhere strictly to the journal’s formatting style.

Reviewer #2: The authors have presented a detailed report on their study to develop a risk assessment indicator system for common diseases in children and adolescents. I have some suggestions :

1. Line 8 : Please make the objective more detailed eg which common diseases have been focused on, here? What are the ages of the subjects under study? What was the overall goal of the study?

**********

6. PLOS authors have the option to publish the peer review history of their article (what does this mean?). If published, this will include your full peer review and any attached files.

If you choose “no”, your identity will remain anonymous but your review may still be made public.

**Do you want your identity to be public for this peer review?**  For information about this choice, including consent withdrawal, please see our Privacy Policy.

Reviewer #1: **Yes:** WDJK Amarasena

Reviewer #2: No

**********

[NOTE: If reviewer comments were submitted as an attachment file, they will be attached to this email and accessible via the submission site. Please log into your account, locate the manuscript record, and check for the action link "View Attachments". If this link does not appear, there are no attachment files.]

To ensure your figures meet our technical requirements, please review our figure guidelines: https://journals.plos.org/plosone/s/figures

You may also use PLOS’s free figure tool, NAAS, to help you prepare publication quality figures: https://journals.plos.org/plosone/s/figures#loc-tools-for-figure-preparation.

NAAS will assess whether your figures meet our technical requirements by comparing each figure against our figure specifications.

# Author comment: Author response to Decision Letter 1

**DOI:** 10.1371/journal.pone.0351870.r002

8 Mar 2026

Response to Reviewers

Dear Editors and Reviewers,

We would like to express our sincere gratitude to you for your valuable time, meticulous review, and constructive comments on our manuscript entitled “A Risk Assessment Indicator System for Common Diseases in Children and Adolescents”. These opinions help to improve academic rigor of our article Based on your suggestion and request,we have made corrected modifications on the revised manuscript. We have carefully considered all the suggestions and made corresponding revisions to improve the quality and rigor of the manuscript. Below is a detailed response to each comment.

1. Please ensure that your manuscript meets PLOS ONE's style requirements, including those for file naming. The PLOS ONE style templates can be found at https://journals.plos.org/plosone/s/file?id=wjVg/PLOSOne_formatting_sample_main_body.pdf and https://journals.plos.org/plosone/s/file?id=ba62/PLOSOne_formatting_sample_title_authors_affiliations.pdf

We have carefully reviewed the PLOS ONE style templates and adjusted the manuscript to fully meet the journal’s formatting requirements, including file naming conventions. All sections, headings, and citations have been standardized in accordance with the provided guidelines.

2. Please provide additional details regarding participant consent. In the ethics statement in the Methods and online submission information, please ensure that you have specified (1) whether consent was informed and (2) what type you obtained (for instance, written or verbal, and if verbal, how it was documented and witnessed). If your study included minors, state whether you obtained consent from parents or guardians. If the need for consent was waived by the ethics committee, please include this information.

In the Ethics Statement section of the Methods, we have supplemented the following details regarding participant consent:

Oral informed consent was obtained from all experts prior to their engagement in the consultation.

The first page of the consultation questionnaire included a formal invitation letter that clearly specified the research objectives, scope of the consultation, and other relevant information. Questionnaires were distributed via email, and completed responses were returned by the same means.

This study employs the Delphi technique for expert consultation with 16 experts. This study did not involve minors. Thus, consent from parents or guardians was not applicable.

3. We note that your Data Availability Statement is currently as follows: All relevant data are within the manuscript and its Supporting Information files.

Please confirm at this time whether or not your submission contains all raw data required to replicate the results of your study.

The original data were not included in the initial submission, but they are complete and available.We will upload the original data as supplementary information file.

4. Thank you for stating the following financial disclosure:

Three-year Action Plan for Strengthening the Construction of Public Health System in Shanghai（2023—2025）（GWVI-11.1-32）

Please state what role the funders took in the study. If the funders had no role, please state: "The funders had no role in study design, data collection and analysis, decision to publish, or preparation of the manuscript."

If this statement is not correct you must amend it as needed.

Please include this amended Role of Funder statement in your cover letter; we will change the online submission form on your behalf.

We have included the Role of Funder statement in the cover letter, with the specific wording as follows: “The funders had no role in study design, data collection and analysis, decision to publish, or preparation of the manuscript.” This revision fully addresses the editor’s requirement.

5. We notice that your supplementary [tables 1, 2 and 3] are included in the manuscript file. Please remove them and upload them with the file type 'Supporting Information'. Please ensure that each Supporting Information file has a legend listed in the manuscript after the references list.

We have prepared corresponding legends for each Supporting Information file (Supplementary Tables 1–3) and included them in the manuscript after the references list as required.

Reviewer #1:

Abstract

• Start each subsection on a new line.

• Add a brief introductory background before the objectives.

• Strengthen the justification briefly.

We sincerely appreciate your constructive suggestion. We have revised the abstract to start each subsection (Background, Objective, Methods, Results, Conclusion) on a new line.

A brief introductory background has been added before the objectives to clarify the significance of constructing the indicator system. The background content is as follows: “Adolescence is a critical stage for healthy development. Currently, common diseases such as myopia, obesity, dental caries, and spinal curvature abnormalities are highly prevalent among children and adolescents, with an obvious trend of younger onset. These diseases are caused by the complex interaction of multi-dimensional factors. However, existing interventions are mostly limited to individual diseases, lacking systematic prevention and control tools. Therefore, there is an urgent need to construct a scientific evaluation index system for health-influencing factors.”

The justification for the study has been strengthened by briefly mentioning the policy, and the revision is as follows: “Guided by China's Outline for Children's Development (2021-2030) and the "Healthy China 2030" Planning Outline, a three-level indicator framework was initially formulated through literature research and expert meetings.”

Introduction

• Please check and standardize the referencing style throughout the manuscript.

• Lines 26: The statement that obesity, myopia, dental caries, and spinal curvature abnormalities are described as “common” requires justification. Please provide prevalence data and improve the flow of supporting evidence.

• Please clarify whether communicable diseases are being considered. If not, specify that the focus is on common Non-Communicable Diseases (NCDs).

• Lines 38: Statements on the immediate and long-term health consequences of childhood and adolescent obesity should be supported with appropriate references and relevant statistics.

• Lines 44: Please explain how the common diseases were identified and selected, and clearly state which diseases were included.

Thank you for your valuable suggestion. We have thoroughly checked and standardized the referencing style throughout the manuscript to adhere to PLOS ONE’s requirements, including consistent formatting of author names, journal titles, and DOI inclusion where available.

Obesity, myopia, dental caries, and spinal curvature abnormalities are explicitly classified as “common diseases” in relevant Chinese government policy documents. To strengthen our argument, we supplemented the corresponding prevalence data. The revised content is as follows: “Accordingly, National Health Commission of the People’s Republic of China has formulated the "Five‑Health" Promotion Action Plan for Children and Adolescents (2026–2030)[1], which identifies obesity, myopia, dental caries, and spinal curvature abnormalities as key priority areas for prevention and control. From the perspective of specific data, according to WHO data in 2022, 390 million children and adolescents aged 5–19 years had overweight, including 160 million with obesity[7]”

We have stated that this study focuses on common non-communicable diseases (NCDs) in children and adolescents explicitly identified in relevant Chinese government policy documents. The revised content is as follows: “The World Health Organization (WHO) has included childhood myopia, dental caries, and obesity in its non‑communicable disease prevention and control framework[2]. Accordingly, National Health Commission of the People’s Republic of China has formulated the "Five‑Health" Promotion Action Plan for Children and Adolescents (2026–2030)[1], which identifies obesity, myopia, dental caries, and spinal curvature abnormalities as key priority areas for prevention and control.”

For the immediate and long-term health consequences of childhood and adolescent obesity, we have added relevant references and statistics. The revised content is as follows: “For example, a large cross-sectional study based on nationally representative data of U.S. children and adolescents showed that, compared with those with mild-to-moderate obesity (body mass index [BMI] ≥ 95th percentile to <160% of the 95th percentile), individuals with extremely severe obesity (BMI ≥160% of the 95th percentile) had a 4.94-fold higher risk of prediabetes/diabetes and a 6.74-fold higher risk of metabolic dysfunction–associated steatotic liver disease[14]. A large life-course cohort study[15] has provided compelling evidence for the long-term health impacts of childhood obesity. Compared with children whose childhood BMI was between the 15th and 50th percentiles, those in the obese group (≥99th percentile) had hazard ratios (HRs) of 2.00 and 1.68 for developing endocrine/metabolic diseases and circulatory system diseases in adulthood.”

The selection of common diseases in this study is primarily based on the explicit definition in China’s "Five-Health" Promotion Action Plan for Children and Adolescents (2026–2030) — this policy classifies myopia, obesity, dental caries, and spinal curvature abnormalities as common diseases requiring priority prevention and control among children and adolescents, hence this study focuses on these four conditions with clear policy orientation.

Methods

• The Ethics section should be placed as the final subsection of the Methods.

• Lines 79: Please describe how the first-, second-, and third-level indicators were developed, including the rationale for classification and the process used to select experts.

We are grateful for your professional suggestion. We have relocated the Ethics Statement to the final subsection of the Methods section, as requested.

We have detailed the development process of first-, second-, and third-level indicators, as well as the expert selection rationale. The revised content is as follows: “

Subjects

Delphi experts were selected based on the following criteria : (1) Professional Field: Experts should have rich practical experience in fields such as Child & Adolescent Health, Maternal and Child Health (MCH), Pediatrics, Preventive Medicine, Health Management, and Nursing. (2) Academic Level: They should possess a relatively high academic level in relevant fields and have a certain professional influence in the industry. (3) Title and Qualifications: They need to hold an associate senior or above professional title, or have an equivalent management rank. (4) Work Experience: They should have at least 11 years of work experience in related fields, with solid industry accumulation and practical knowledge. Potential experts were initially identified through recommendations from collaborative institutions and peers. After eligibility verification, consultation questionnaires were sent via email. Ultimately, a total of 16 experts from Centers for Disease Control and Prevention, universities, hospitals, maternal and child health centers, health commissions, primary/secondary schools, and community health service centers were enrolled in this study.

Method Preliminary index system

The first-level and second-level indicators were classified based on national policies and also with reference to the life course perspective-based adolescent health assessment framework proposed by Tao FB (2014)[17]. Guided by core policy documents including the National Program for Child Development (2021-2030) and the Healthy China 2030 Plan Outline, this study integrated the life course perspective and adolescent health conceptual framework to divide the content of children and adolescents' health monitoring into 4 first-level indicators and 8 second-level indicators. The construction of third-level indicators was mainly based on literature review, existing monitoring systems, and the needs of expert consensus. A systematic search was conducted across Chinese and English databases (e.g., CNKI, Wanfang Data, PubMed) and official government websites, using keywords such as "children", "adolescents", "students", "myopia", "overweight", "obesity", "dental caries", "spinal curvature abnormalities", "Shanghai", "intervention", "management", "evaluation", "Myopia", "Students", "Adolescents", "Children", "Oral", "Scoliosis", "Caries", "Shanghai", and "Obesity". By incorporating mature indicators from the Shanghai Student Common Diseases and Health Influencing Factors Monitoring and Intervention Scale, a total of 54 third-level indicators were finally formulated.

Form a pool of index items

Building on these preliminary efforts, the research team integrated policy requirements, theoretical frameworks, literature evidence, and existing monitoring indicators to establish an evaluation indicator inventory comprising 4 first-level indicators, 8 second-level indicators, and 54 third-level indicators. This inventory covers health determinants, health status, and relevant health work related to overweight and obesity, myopia, dental caries, and spinal curvature abnormalities in children and adolescents. Based on this repository, an expert consultation questionnaire was designed for subsequent Delphi method implementation or expert consultation procedures.”

Discussion

• Add a brief summary of the key findings at the beginning of the Discussion section.

References

• Please include DOIs where available and ensure that all references adhere strictly to the journal’s formatting style.

We are grateful for your professional comment. We have added a brief summary of key findings at the beginning of the Discussion section. The revised statement reads as follows: “Based on a life-course perspective and the conceptual framework of adolescent health[18], this study constructed a risk assessment indicator system for four highly prevalent common diseases (myopia, obesity, dental caries, and spinal curvature abnormalities) in children and adolescents aged 7–19 years, using the Delphi method with two rounds of expert consultation. The final system comprises 55 three-level indicators across five dimensions: social determinants, school environment, health services, genetic and early life factors, and health-related behavioral factors.”

We have supplemented DOIs for all applicable references and standardized the formatting to comply with PLOS ONE’s reference style. Some Chinese core journal articles do not have an official DOI.

Reviewer #2: The authors have presented a detailed report on their study to develop a risk assessment indicator system for common diseases in children and adolescents. I have some suggestions :

1. Line 8 : Please make the objective more detailed eg which common diseases have been focused on, here? What are the ages of the subjects under study? What was the overall goal of the study?

We are grateful for your professional comment. We have made the objective more detailed by clarifying the target diseases, subject age range, and overall goal. The revised statement reads as follows: “This study focuses on children and adolescents aged 7-19 years, with a specific emphasis on four highly prevalent common diseases: myopia, obesity, dental caries, and spinal curvature abnormalities. It aims to construct a three-level evaluation indicator system covering multi-dimensional health-influencing factors, providing a standardized and operable evaluation tool for the comprehensive prevention and control of common diseases in children and adolescents as well as the formulation of relevant policies, ultimately improving the health status of the child and adolescent population.”

Once again, we would like to thank the Editors and Reviewers for their valuable comments and suggestions. We believe these revisions have significantly improved the quality and clarity of the manuscript. We hope the revised version meets the publication standards of PLOS ONE and look forward to your further feedback.

Attachment Submitted filename: Response to Reviewers.docx

# Peer review: Decision Letter 1

**DOI:** 10.1371/journal.pone.0351870.r003

**Authors:** Rajiv Janardhanan

8 May 2026

PONE-D-25-58634R1

A Risk Assessment Indicator System for Common Diseases in Children and Adolescents

PLOS One

Dear Dr. Liang,

Thank you for submitting your manuscript to PLOS ONE. After careful consideration, we feel that it has merit but does not fully meet PLOS ONE’s publication criteria as it currently stands. Therefore, we invite you to submit a revised version of the manuscript that addresses the points raised during the review process.

Please submit your revised manuscript by Jun 22 2026 11:59PM. If you will need more time than this to complete your revisions, please reply to this message or contact the journal office at plosone@plos.org. When you're ready to submit your revision, log on to https://www.editorialmanager.com/pone/ and select the 'Submissions Needing Revision' folder to locate your manuscript file.

Please include the following items when submitting your revised manuscript:

- A letter that responds to each point raised by the academic editor and reviewer(s). You should upload this letter as a separate file labeled 'Response to Reviewers'.
- A marked-up copy of your manuscript that highlights changes made to the original version. You should upload this as a separate file labeled 'Revised Manuscript with Track Changes'.
- An unmarked version of your revised paper without tracked changes. You should upload this as a separate file labeled 'Manuscript'.

If you would like to make changes to your financial disclosure, please include your updated statement in your cover letter. Guidelines for resubmitting your figure files are available below the reviewer comments at the end of this letter.

If applicable, we recommend that you deposit your laboratory protocols in protocols.io to enhance the reproducibility of your results. Protocols.io assigns your protocol its own identifier (DOI) so that it can be cited independently in the future. For instructions see: https://journals.plos.org/plosone/s/submission-guidelines#loc-laboratory-protocols. Additionally, PLOS ONE offers an option for publishing peer-reviewed Lab Protocol articles, which describe protocols hosted on protocols.io. Read more information on sharing protocols at https://plos.org/protocols?utm_medium=editorial-email&utm_source=authorletters&utm_campaign=protocols.

As the corresponding author, your ORCID iD is verified in the submission system and will appear in the published article. PLOS supports the use of ORCID, and we encourage all coauthors to register for an ORCID iD and use it as well. Please encourage your coauthors to verify their ORCID iD within the submission system before final acceptance, as unverified ORCID iDs will not appear in the published article. *Only*  the individual author can complete the verification step; PLOS staff *cannot*  verify ORCID iDs on behalf of authors.

We look forward to receiving your revised manuscript.

Kind regards,

Rajiv Janardhanan, Ph.D.

Academic Editor

PLOS One

Journal Requirements:

If the reviewer comments include a recommendation to cite specific previously published works, please review and evaluate these publications to determine whether they are relevant and should be cited. There is no requirement to cite these works unless the editor has indicated otherwise.

Please review your reference list to ensure that it is complete and correct. If you have cited papers that have been retracted, please include the rationale for doing so in the manuscript text, or remove these references and replace them with relevant current references. Any changes to the reference list should be mentioned in the rebuttal letter that accompanies your revised manuscript. If you need to cite a retracted article, indicate the article’s retracted status in the References list and also include a citation and full reference for the retraction notice.

Additional Editor Comments:

Requesting the authors to kindly respond to Reviewer 3's comments.

[Note: HTML markup is below. Please do not edit.]

Reviewers' comments:

Reviewer's Responses to Questions

**Comments to the Author**

1. If the authors have adequately addressed your comments raised in a previous round of review and you feel that this manuscript is now acceptable for publication, you may indicate that here to bypass the “Comments to the Author” section, enter your conflict of interest statement in the “Confidential to Editor” section, and submit your "Accept" recommendation.

Reviewer #2: All comments have been addressed

Reviewer #3: (No Response)

**********

2. Is the manuscript technically sound, and do the data support the conclusions?

The manuscript must describe a technically sound piece of scientific research with data that supports the conclusions. Experiments must have been conducted rigorously, with appropriate controls, replication, and sample sizes. The conclusions must be drawn appropriately based on the data presented.

Reviewer #2: Yes

Reviewer #3: Partly

**********

3. Has the statistical analysis been performed appropriately and rigorously?

Reviewer #2: Yes

Reviewer #3: Yes

**********

4. Have the authors made all data underlying the findings in their manuscript fully available?

The PLOS Data policy requires authors to make all data underlying the findings described in their manuscript fully available without restriction, with rare exception (please refer to the Data Availability Statement in the manuscript PDF file). The data should be provided as part of the manuscript or its supporting information, or deposited to a public repository. For example, in addition to summary statistics, the data points behind means, medians and variance measures should be available. If there are restrictions on publicly sharing data—e.g. participant privacy or use of data from a third party—those must be specified.

Reviewer #2: Yes

Reviewer #3: Yes

**********

5. Is the manuscript presented in an intelligible fashion and written in standard English?

PLOS ONE does not copyedit accepted manuscripts, so the language in submitted articles must be clear, correct, and unambiguous. Any typographical or grammatical errors should be corrected at revision, so please note any specific errors here.

Reviewer #2: Yes

Reviewer #3: No

**********

6. Review Comments to the Author

Please use the space provided to explain your answers to the questions above. You may also include additional comments for the author, including concerns about dual publication, research ethics, or publication ethics. (Please upload your review as an attachment if it exceeds 20,000 characters)

Reviewer #2: (No Response)

Reviewer #3: The authors have presented a detailed report on their study to develop a risk assessment indicator system for common diseases in children and adolescents. It is indeed a very novel approach to improve the health outcomes. I have a few suggestions that could help in the betterment of the manuscript.

1. The Objective section of the Abstract is too long. The authors need to make the objective section of the manuscript briefer.

2. The authors must explain clearly and properly how the common diseases were identified and selected.

3. Line 40 - The term “Keywords” must be written properly.

4. Line 50 - There are multiple syntax errors in the manuscript. The authors must reframe the manuscript by reducing the syntax errors.

5. The authors must also try to mention the prevalence and incidence rates of all the most common diseases in the Introduction section of the manuscript.

6. Some authors must also clearly describe how the ethnic differences between the subjects in the manuscript are so that the race-specificities, age-specificities, or sex-specificities can be clearly depicted in the manuscript.

7. It is suggested that the authors elaborate on the key results in the Discussion section of the manuscript.

8. Multiple references do not have a DOI. Kindly add DOIs to all references in the manuscript, including references 6, 8, 17, 18, 19, 20, and 21.

9. The authors are requested to maintain the same reference style throughout the manuscript.

**********

7. PLOS authors have the option to publish the peer review history of their article (what does this mean?). If published, this will include your full peer review and any attached files.

If you choose “no”, your identity will remain anonymous but your review may still be made public.

**Do you want your identity to be public for this peer review?**  For information about this choice, including consent withdrawal, please see our Privacy Policy.

Reviewer #2: No

Reviewer #3: **Yes:** Tridip Mitra

**********

[NOTE: If reviewer comments were submitted as an attachment file, they will be attached to this email and accessible via the submission site. Please log into your account, locate the manuscript record, and check for the action link "View Attachments". If this link does not appear, there are no attachment files.]

To ensure your figures meet our technical requirements, please review our figure guidelines: https://journals.plos.org/plosone/s/figures

You may also use PLOS’s free figure tool, NAAS, to help you prepare publication quality figures: https://journals.plos.org/plosone/s/figures#loc-tools-for-figure-preparation.

NAAS will assess whether your figures meet our technical requirements by comparing each figure against our figure specifications.

Attachment Submitted filename: Plosone comment.docx

# Author comment: Author response to Decision Letter 2

**DOI:** 10.1371/journal.pone.0351870.r004

18 May 2026

Dear Editors and Reviewers,

We would like to express our sincere gratitude to you for your valuable time, meticulous review, and constructive comments on our manuscript entitled “A Risk Assessment Indicator System for Common Diseases in Children and Adolescents”. These opinions help to improve academic rigor of our article Based on your suggestion and request,we have made corrected modifications on the revised manuscript. We have carefully considered all the suggestions and made corresponding revisions to improve the quality and rigor of the manuscript. Below is a detailed response to each comment.

1.The Objective section of the Abstract is too long. The authors need to make the objective section of the manuscript briefer.

Thank you very much for your helpful suggestion. We have condensed and streamlined the objective section of the abstract, removing redundant descriptions and keeping only core research purposes. The revised statement reads as follows: Targeting the four key common diseases emphasized in China’s national health policies - myopia, obesity, dental caries, and spinal curvature abnormalities - among children and adolescents aged 7 - 19 years, this study constructed a multi-dimensional risk assessment indicator system to support evidence-based prevention and policy-making for this population.

2.The authors must explain clearly and properly how the common diseases were identified and selected.

Thank you very much for your helpful suggestion. We explain in the Introduction that the four common diseases (myopia, obesity, dental caries, and spinal curvature abnormalities) were selected because they are the key priority diseases identified in China’s national child and adolescent health policies, including the “Five-Health” Promotion Action Plan for Children and Adolescents (2026 – 2030). These diseases pose a serious health burden and have been emphasized by the World Health Organization, so they were selected as the core diseases for this system.

3.Line 40 - The term “Keywords” must be written properly.

Thank you for pointing out this mistake. We have properly revised the writing form of "Keywords" as required.

4.Line 50 - There are multiple syntax errors in the manuscript. The authors must reframe the manuscript by reducing the syntax errors.

We highly appreciate your careful review. We have thoroughly checked and revised the full manuscript, rephrased inappropriate expressions, and corrected existing syntax errors to ensure fluent and standard academic writing.

5.The authors must also try to mention the prevalence and incidence rates of all the most common diseases in the Introduction section of the manuscript.

We fully agree with this suggestion. Relevant data on spinal curvature abnormalities have been supplemented in the introduction section. The revised statement reads as follows: A provincial surveillance study in Jiangsu, China reported an overall detection rate of 2.1% for spinal curvature abnormalities among children and adolescents during 2021 - 2023, highlighting this condition as an important public health issue.

6.Some authors must also clearly describe how the ethnic differences between the subjects in the manuscript are so that the race-specificities, age-specificities, or sex-specificities can be clearly depicted in the manuscript.

Thank you very much for your helpful suggestion. All participants in our study are Chinese without ethnic differences.

7.It is suggested that the authors elaborate on the key results in the Discussion section of the manuscript.

Thank you for your constructive suggestion. We have elaborated on the key results in the Discussion section. The revised statement reads as follows:

Based on a life-course perspective and the conceptual framework of adolescent health[18], this study constructed a risk assessment indicator system for four highly prevalent common diseases (myopia, obesity, dental caries, and spinal curvature abnormalities) in children and adolescents aged 7–19 years, using the Delphi method with two rounds of expert consultation. The final system comprises 55 three-level indicators across five dimensions: social determinants, school environment, health services, genetic and early life factors, and health-related behavioral factors. The results of two rounds of expert consultation showed that the questionnaire response rate was 100.0%. The mean familiarity coefficient (Cs), judgment coefficient (Ca) and authority coefficient (Cr) of experts were 0.83, 0.93 and 0.88. Expert agreement improved from round 1 to round 2 and reached statistical significance.

This study shifts the paradigm from single-disease monitoring to a shared risk factor-based assessment for obesity, myopia, dental caries, and spinal curvature abnormalities in children and adolescents. Including school environment, health-related behavioral factors, and genetic and early-life factors, the indicator system extends the traditional student health monitoring framework. This indicator system provides an evidence base for screening, referral, and prioritization of interventions.

However, this study also has certain limitations. Some indicators (such as "average daily screen time in infancy")rely on parental recall, making the data susceptible to recall bias. Therefore, future validation using birth cohort studies is necessary. Regional differences have not been fully considered. For example, the feasibility of "winter and summer sports homework assignment rate" is poor in rural areas. In the future, it is necessary to formulate adaptability standards for different regions. The indicator system was developed in the context of Shanghai’s student health monitoring practice and based on a Chinese expert panel; therefore, its applicability to other regions requires further validation. Furthermore, the indicator framework established in this study comprises 55 indicators. While comprehensive, the large number of indicators may compromise practicality and cost-effectiveness in large-scale monitoring. Future efforts should focus on developing a streamlined core indicator set that retains analytical robustness while enhancing feasibility across diverse implementation scenarios. Finally, the index system needs to verify the validity in monitoring practice.

8.Multiple references do not have a DOI. Kindly add DOIs to all references in the manuscript, including references 6, 8, 17, 18, 19, 20, and 21.

Thank you for pointing this out. We have added the missing DOIs to all references.

9.The authors are requested to maintain the same reference style throughout the manuscript.

Thank you very much for your helpful suggestion. We have unified and standardized the citation format of all references in manuscript, ensuring consistent reference style throughout the paper.

Attachment Submitted filename: Response_to_Reviewers_auresp_2.docx

# Peer review: Decision Letter 2

**DOI:** 10.1371/journal.pone.0351870.r005

**Authors:** Rajiv Janardhanan

2 Jun 2026

A Risk Assessment Indicator System for Common Diseases in Children and Adolescents

PONE-D-25-58634R2

Dear Dr. Liang,

We’re pleased to inform you that your manuscript has been judged scientifically suitable for publication and will be formally accepted for publication once it meets all outstanding technical requirements.

Within one week, you’ll receive an e-mail detailing the required amendments. When these have been addressed, you’ll receive a formal acceptance letter and your manuscript will be scheduled for publication.

An invoice will be generated when your article is formally accepted. Please note, if your institution has a publishing partnership with PLOS and your article meets the relevant criteria, all or part of your publication costs will be covered. Please make sure your user information is up-to-date by logging into Editorial Manager at Editorial Manager® and clicking the ‘Update My Information' link at the top of the page. For questions related to billing, please contact billing support.

If your institution or institutions have a press office, please notify them about your upcoming paper to help maximize its impact. If they’ll be preparing press materials, please inform our press team as soon as possible -- no later than 48 hours after receiving the formal acceptance. Your manuscript will remain under strict press embargo until 2 pm Eastern Time on the date of publication. For more information, please contact onepress@plos.org.

Kind regards,

Rajiv Janardhanan, Ph.D.

Academic Editor

PLOS One

Additional Editor Comments (optional):

Reviewers' comments:

Reviewer's Responses to Questions

**Comments to the Author**

1. If the authors have adequately addressed your comments raised in a previous round of review and you feel that this manuscript is now acceptable for publication, you may indicate that here to bypass the “Comments to the Author” section, enter your conflict of interest statement in the “Confidential to Editor” section, and submit your "Accept" recommendation.

Reviewer #3: All comments have been addressed

**********

2. Is the manuscript technically sound, and do the data support the conclusions?

The manuscript must describe a technically sound piece of scientific research with data that supports the conclusions. Experiments must have been conducted rigorously, with appropriate controls, replication, and sample sizes. The conclusions must be drawn appropriately based on the data presented.

Reviewer #3: Yes

**********

3. Has the statistical analysis been performed appropriately and rigorously?

Reviewer #3: Yes

**********

4. Have the authors made all data underlying the findings in their manuscript fully available?

The PLOS Data policy requires authors to make all data underlying the findings described in their manuscript fully available without restriction, with rare exception (please refer to the Data Availability Statement in the manuscript PDF file). The data should be provided as part of the manuscript or its supporting information, or deposited to a public repository. For example, in addition to summary statistics, the data points behind means, medians and variance measures should be available. If there are restrictions on publicly sharing data—e.g. participant privacy or use of data from a third party—those must be specified.

Reviewer #3: Yes

**********

5. Is the manuscript presented in an intelligible fashion and written in standard English?

PLOS ONE does not copyedit accepted manuscripts, so the language in submitted articles must be clear, correct, and unambiguous. Any typographical or grammatical errors should be corrected at revision, so please note any specific errors here.

Reviewer #3: Yes

**********

6. Review Comments to the Author

Please use the space provided to explain your answers to the questions above. You may also include additional comments for the author, including concerns about dual publication, research ethics, or publication ethics. (Please upload your review as an attachment if it exceeds 20,000 characters)

Reviewer #3: The manuscript is well written, scientifically sound, and suitable for publication in its current form. I recommend its acceptance and consider the review process complete.

**********

7. PLOS authors have the option to publish the peer review history of their article (what does this mean?). If published, this will include your full peer review and any attached files.

If you choose “no”, your identity will remain anonymous but your review may still be made public.

**Do you want your identity to be public for this peer review?**  For information about this choice, including consent withdrawal, please see our Privacy Policy.

Reviewer #3: **Yes:** Tridip Mitra

**********

# Editor report: Acceptance letter

**DOI:** 10.1371/journal.pone.0351870.r006

**Authors:** Rajiv Janardhanan

PONE-D-25-58634R2

PLOS One

Dear Dr. Liang,

I'm pleased to inform you that your manuscript has been deemed suitable for publication in PLOS One. Congratulations! Your manuscript is now being handed over to our production team.

At this stage, our production department will prepare your paper for publication. This includes ensuring the following:

* All references, tables, and figures are properly cited

* All relevant supporting information is included in the manuscript submission,

* There are no issues that prevent the paper from being properly typeset

You will receive further instructions from the production team, including instructions on how to review your proof when it is ready. Please keep in mind that we are working through a large volume of accepted articles, so please give us a few days to review your paper and let you know the next and final steps.

Lastly, if your institution or institutions have a press office, please let them know about your upcoming paper now to help maximize its impact. If they'll be preparing press materials, please inform our press team within the next 48 hours. Your manuscript will remain under strict press embargo until 2 pm Eastern Time on the date of publication. For more information, please contact onepress@plos.org.

You will receive an invoice from PLOS for your publication fee after your manuscript has reached the completed accept phase. If you receive an email requesting payment before acceptance or for any other service, this may be a phishing scheme. Learn how to identify phishing emails and protect your accounts at https://explore.plos.org/phishing.

If we can help with anything else, please email us at customercare@plos.org.

Thank you for submitting your work to PLOS ONE and supporting open access.

Kind regards,

PLOS ONE Editorial Office Staff

on behalf of

Dr. Rajiv Janardhanan

Academic Editor

PLOS One
